# Identification and metabolite profiling of alkaloids in aerial parts of *Papaver rhoeas* by liquid chromatography coupled with quadrupole time‐of‐flight tandem mass spectrometry

**DOI:** 10.1002/jssc.201701402

**Published:** 2018-04-25

**Authors:** Jae‐Hyeon Oh, In Jin Ha, Min Young Lee, Eun‐Ok Kim, Dain Park, Jun‐Hee Lee, Seok‐Geun Lee, Do‐Wan Kim, Tae‐Ho Lee, Eui‐Ju Lee, Chang‐Kug Kim

**Affiliations:** ^1^ Genomics Division Department of Agricultural Biotechnology National Institute of Agricultural Science (NAS) Rural Development Administration (RDA) Jeollabuk‐do Republic of Korea; ^2^ Korean Medicine Clinical Trial Center (K‐CTC) Kyung Hee University Korean Medicine Hospital Seoul Republic of Korea; ^3^ KHU‐KIST Department of Converging Science & Technology Kyung Hee University Seoul Republic of Korea; ^4^ Department of Sasang Constitutional Medicine College of Korean Medicine Kyung Hee University Seoul Republic of Korea

**Keywords:** alkaloids, liquid chromatography, metabolite profiling, *Papaver rhoeas*, *Papaver somniferum*

## Abstract

*Papaver* plants can produce diverse bioactive alkaloids. *Papaver rhoeas* Linnaeus (common poppy or corn poppy) is an annual flowering medicinal plant used for treating cough, sleep disorder, and as a sedative, pain reliever, and food. It contains various powerful alkaloids like rhoeadine, benzylisoquinoline, and proaporphine. To investigate and identify alkaloids in the aerial parts of *P. rhoeas*, samples were collected at different growth stages and analyzed using liquid chromatography coupled with quadrupole time‐of‐flight tandem mass spectrometry. A liquid chromatography with mass spectrometry method was developed for the identification and metabolite profiling of alkaloids for *P. rhoeas* by comparing with *Papaver somniferum*. Eighteen alkaloids involved in benzylisoquinoline alkaloid biosynthesis were used to optimize the liquid chromatography gradient and mass spectrometry conditions. Fifty‐five alkaloids, including protoberberine, benzylisoquinoline, aporphine, benzophenanthridine, and rhoeadine‐type alkaloids, were identified authentically or tentatively by liquid chromatography coupled with quadrupole time‐of‐flight tandem mass spectrometry in samples taken during various growth stages. Rhoeadine alkaloids were observed only in *P. rhoeas* samples, and codeine and morphine were tentatively identified in *P. somniferum*. The liquid chromatography coupled with quadrupole time‐of‐flight tandem mass spectrometry method can be a powerful tool for the identification of diverse metabolites in the genus *Papaver*. These results may help understand the biosynthesis of alkaloids in *P. rhoeas* and evaluate the quality of this plant for possible medicinal applications.

**Article‐Related Abbreviations**
BIA: benzylisoquiquinoline alkaloid
PS: *P. somniferum* seeds
RA: WS2981 *P. rhoeas* seeds from Hoengseong in the Province of Gangwon, South Korea
RS: Shiri *P. rhoeas* seeds from Moscow, Russia
RT: retention time

## 1 INTRODUCTION

The annual flowering plant *Papaver rhoeas* L. (red poppy or corn poppy), is a species of the poppy family, Papaveraceae. It has been used in folk medicine for the treatment of inflammation, cough, diarrhea, respiratory problems, asthma, insomnia, and pain, and it can also be consumed as food 1, 2, 3, 4, 5. Various phytochemical components can be found in corn poppy, such as alkaloids 2, 6, 7, 8, anthocyanins 9, flavonoids 10, and essential oils 11. *P. rhoeas* can be cultivated in regions with a temperate climate. In Korea, it is widely distributed and is grown as an ornamental plant. This plant is sometimes mistaken for *Papaver somniferum* L. (PS), the strictly controlled opium poppy, containing narcotic substances such as morphine, codeine, and the baine 12. Plants in the genus *Papaver* can produce diverse alkaloids, mainly in the form of benzylisoquiquinoline alkaloids (BIAs) 13, 14. Alkaloids from *Papaver* species possess significant biological activity 13, 15, and the alkaloid content of *Papaver* plants varies greatly depending on their growth stage, conditions, and origin 4, 12, 16. Rapid and reliable analytical techniques must be developed to facilitate identification and structural determination of alkaloids in complex extracts and medicine herbs 17, 18, 19, 20, 21, 22, 23. To discover medicinally relevant compounds and potential alternatives to plants as the commercial source of valuable BIAs, metabolomic studies of the *Papaver* species have been reported by metabolomics with chemometric approaches and transcript profiling 13, 15, 24, 25, 26. However, to date, most studies have focused on the opium poppy and there have been few analytical studies on the alkaloids in *P. rhoeas* 4, 12. This is despite various reports on biological activity of its extracts based on both in vitro and in vivo experiments and clinical studies 4, 5, 7, 27. Many alkaloids likely present in this plant are yet to be identified. Therefore, investigation and identification of known and unknown alkaloids in *P. rhoeas* is needed for pharmacological studies, plant extract quality evaluations, and to understand species‐specific biosynthetic metabolism. This study aims to develop an analytical method based on LC–QTOF‐MS/MS for metabolite profiling and identification of alkaloids in *P. rhoeas* in a single LC run. Two types of *P. rhoeas* with different origins and one *P. somniferum* were cultivated and collected at different growth stages to measure the growth stage dependent variation of metabolites and to limit environmental influences. Samples collected at different time points were analyzed to achieve a more comprehensive understanding of BIA biosynthesis. The aim of the present study, in addition to developing novel LC–QTOF‐MS/MS methods, was to investigate alkaloids and their changes in *P. rhoeas* at different growth stages.

## 2 MATERIALS AND METHODS

### 2.1 Plant material

The aerial parts of *P. rhoeas* harvested at three different growth stages were provided by the National Academy of Agricultural Science, Rural Development Administration (Korea). The specimens used in this study were deposited in the Genomics Division of the National Academy of Agricultural Science. The two types of *P. rhoeas* seeds were of different origins; one (resource name: Shiri) originated from Moscow, Russia (RS) and the other (resource name: WS2981) originated from Hoengseong in the Province of Gangwon, South Korea (RA). The seeds were used after disinfection according to a method described elsewhere 28, and pre‐processing with low temperature treatment at 4°C for 90 days 29. Two types of *P. rhoeas* (RS and RA) and one type of *P. somniferum* (PS) were sown in April. The growth conditions were 26°C and relative humidity of 45% under natural light conditions in a glass greenhouse at the NAAS (latitude [N 35° 49′ 53'']; longitude [E 127° 3′ 48″]) in the Republic of Korea. For the *Papaver* genus, the floral axis is observed at 60 days, and flower closing and fruit‐bearing occurs 90 days after germination. Hence, samples were collected at 30, 60, and 90 days.

### 2.2 Reagents and chemicals

Eighteen BIAs were purchased as authentic standards and are listed in Table 1. The reference standard of demetylcoclaurine (20 mg; purity ≥ 98%) and tetrahydropapverine (10 mg; purity ≥ 98%) were purchased from ALB technology (Mongkok Kowloon, Hong Kong). Coclaurine (10 mg; purity ≥ 97.2%), corytuberine (10 mg; purity ≥ 98%), tetrahydroberberin (20 mg; purity ≥ 98%), berberine (20 mg; purity ≥ 98%), stylopine (10 mg; purity ≥ 98.3%), dihydrosanguinarine (10 mg; purity ≥ 98%), sanguinarine (20 mg; purity ≥ 99.8%), protopine (20 mg; purity ≥ 98.3%), and dihydroberberine (10 mg; purity ≥ 98%) were purchased from Chemface (Wuhan, China). (*S*)‐Reticuline (10 mg; purity ≥ 98%) and l‐reticule (10 mg; purity ≥ 98%) were purchased from Carbosynth (Berkshire, UK). Tetrahydrocolumbamine (10 mg; purity ≥ 98%) and scoulerine (10 mg; purity ≥ 98%) were purchased from ChemNorm biotech (Wuhan, China). l‐Tetrahydropalmatine (20 mg; purity ≥ 98%) and allocryptopine (20 mg; purity ≥ 98%) were purchased from Biopurify phytochemicals (Chengdu, China). Chelidonine (10 mg; purity ≥ 97%) was purchased from Sigma–Aldrich (St. Louis, MO). Acetonitrile (ACN) and methanol (MeOH) were purchased from Honeywell Burdick & Jackson (Morristown, NJ) and were of HPLC grade. Analytical‐grade formic acid (99% purity) and ammonium formate were obtained from Sigma–Aldrich. Deionized water (>18 mΩ) was obtained by a pure water purification system (Human, Korea).

**Table 1:** Retention time (RT), MS data, and BIA structural subgroups of the authentic alkaloids used in this study

**Peak no**.	**Alkaloid name**	**RT (min)**	**Formula**	**Adduct**	**Observed mass (Da)**	**Mass error (ppm)**	**BIA structural subgroups derived from the basic benzylisoquinoline subunit**
1	DL‐Demethylcoclaurine	4.98	C_16_H_17_NO_3_	+H	272.1281	−0.1	Benzylsioquinoline
2	Coclaurine	6.01	C_17_H_19_NO_3_	+H	286.1438	0.8	Benzylsioquinoline
3	Tetrahydropapaverine	9.10	C_20_H_25_NO_4_	+H	344.1856	1.0	Benzylsioquinoline
4	*S*‐Reticuline	6.72	C_19_H_23_NO_4_	+H	330.1700	0.8	Benzylsioquinoline
5	Corytuberine	6.29	C_19_H_21_NO_4_	+H	328.1543	0.8	Aporphine
6	l‐Reticuline	6.72	C_19_H_23_NO_4_	+H	330.1700	0.8	Benzylsioquinoline
7	Tetrahydrocolumbamine	8.37	C_20_H_23_NO_4_	+H	342.1700	0.1	Protoberberine (tetrahydroprotoberberine)
8	Scoulerine	6.57	C_19_H_21_NO_4_	+H	328.1543	0.4	Protoberberine (tetrahydroprotoberberine)
9	l‐Tetrahydropalmatine	10.51	C_21_H_25_NO_4_	+H	356.1856	0.9	Protoberberine (tetrahydroprotoberberine)
10	Tetrahydroberberine (canadine)	11.64	C_20_H_21_NO_4_	+H	340.1543	0.8	Protoberberine (tetrahydroprotoberberine)
11	Berberine	13.80	C_20_H_18_NO_4_		336.1236	−0.6	Protoberberine
12	Stylopine	10.85	C_19_H_17_NO_4_	+H	324.1230	0.9	Protoberberine (tetrahydroprotoberberine)
13	Dihydrosanguinarine	19.10	C_20_H_15_NO_4_	+H	334.1074	1.4	Benzophenanthridine
14	Sanguinarine	12.75	C_20_H_14_NO_4_		332.0923	−0.6	Benzophenanthridine
15	Protopine	9.22	C_20_H_19_NO_5_	+H	354.1336	0.8	Protopine
16	Allocryptopine	10.21	C_21_H_23_NO_5_	+H	370.1649	0.1	Protopine
17	Chelidonine	10.18	C_20_H_19_NO_5_	+H	354.1336	−0.1	Benzophenanthridine
18	Dihydroberberine	10.95	C_20_H_19_NO_4_	+H	338.1387	0.1	Protoberberine

### 2.3 Sample preparation

Aerial parts of the lyophilized *Papaver* species were first ground into a fine powder. Approximately 2 g of each sample was then ultrasonicated in 5 mL of ethanol for 30 min, and centrifuged at 13 000 rpm for 15 min at 4°C. The supernatants were filtered through a 0.2 μm polytetrafluoroethylene syringe filter (Thermo Scientific, Waltham, MA). Finally, the filtrate was diluted with ethanol to 20 mg/mL and transferred to an LC sample vial before use.

### 2.4 LC and MS analysis conditions

The LC–MS system consisted of a Thermo Scientific Vanquish UHPLC system (Thermo Fisher Scientific, Sunnyvale, CA) with an Acquity UPLC HSS T3 column (2.1 × 100 mm, 1.7 μm; Waters, Milford, MA) and a Triple TOF 5600^+^ mass spectrometer system (Triple TOF MS; QTOF, Sciex, Foster City, CA). The QTOF‐MS, equipped with a Duospray™ ion source, was used to complete the high‐resolution experiment. The LC gradient used a mobile phase A containing 0.05% formic acid and 2.5 mM ammonium formate in water and a mobile phase B of acetonitrile. The flow rate was kept constant at 0.4 mL/min and the injection volume was 1 μL. The gradient elution system began at 1% B for 2.5 min, 1 to 10% B from 2.5 to 3.0 min, 10 to 19% B from 3.0 to 6.0 min, 19 to 22% from 6.0 to 9.0 min, 22 to 25% B from 9.0 to 14.0 min, 25 to 70% B from 14.0 to 17.0 min, and then increased to 100% B at 19.0 min, held at 100% B for 3 min and then returned to the initial conditions for re‐equilibration.

Mass data acquisition was performed with a Triple TOF 5600^+^ in positive ion mode using the following parameters: source temperature was set at 450°C with a curtain gas flow of 25 L/min (GS1 and GS2 both 50), the ion spray voltage was set at 4500 V, declustering potential was 50 V, and the collision energy was 10 V. High‐purity nitrogen gas was used for the nebulizer/Duospray™ and curtain gases. The QTOF and information‐dependent acquisition scan was operated with a mass range of 50 to 1500 *m/z*. Precursor and product ion calibration were performed in both high sensitivity and high‐resolution modes using a calibrant delivery system before analysis.

Data acquisition and processing were carried out using Analyst TF 1.7, PeakVeiw 2.2, MarterView, and MarkerView 1.2 software (Sciex).

## 3 RESULTS AND DISCUSSION

### 3.1 Sample preparation

Five different extraction solvents were examined for effectiveness in metabolite extraction from the aerial parts of plants: 50 and 80% methanol in water, pure methanol, pure ethanol, and ethyl ether with 10% ammonia. Samples were extracted and analyzed by the LC–QTRAP (hybrid triple quadrupole/linear ion trap) MS system (ABSCIEX, Foster City, CA). To study the effects of the extraction process on metabolite quantitation, the peak intensities of the extracted metabolites were compared. As a first approximation, a gradient system developed in a previous study was used 30. The protopine authentic metabolite was detected in all tested samples. When ethyl ether was used, the peak area of protopine was the highest of all the extraction solvents tested, as shown in Supporting information Table S1 and Figure S1. According to the peak areas, the efficiency of extraction solvents was as follows: ethyl ether with 10% ammonia > pure ethanol > 80% methanol in water > methanol > 50% methanol in water. However, ethyl ether is volatile, and its extracts require additional sample preparation steps including evaporation and re‐dilution to obtain reproducible data. For metabolomics, sample preparation should be easy, convenient, robust, and reproducible, especially for large‐scale analyses. Therefore, ethanol, the second most efficient extraction solvent was selected as the ideal extraction solvent for the aerial parts of *P. rhoeas* and *P. somniferum*.

### 3.2 LC–MS analysis

**Figure 1:**
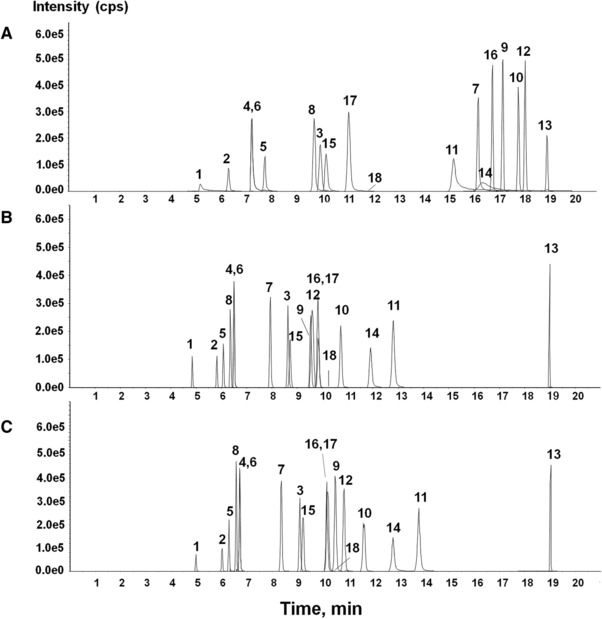
Representative extracted ion chromatography (XIC) of 18 authentic standards with different mobile phase compositions: 5 mM ammonium formate in water/acetonitrile (A), 0.1% formic acid in water/acetonitrile (B), 2.5 mM ammonium formate and 0.05% formic acid in water/acetonitrile (C); (1) dl‐demethylcoclaurine, (2) coclaurine, (3) tetrahydropapaverine, (4) *S*‐reticuline, (5) corytuberine, (6) l‐reticuline, (7) tetrahydrocolumbamine, (8) scoulerine, (9) l‐tetrahydropalmatine, (10) tetrahydroberberine, (11) berberine, (12) stylopine, (13) dihydrosanguinarine, (14) sanguinarine, (15) protopine, (16) chelidonine, (17) allocryptopine, and (18) dihydroberberine

Several previous studies analyzed and separated BIAs using similar analytical techniques as presented here 17, 18, 19, 30, 31, 32. The simultaneous analysis of the alkaloids with different skeletons present in the *Papaver* genus is not a simple task. The chromatographic retention behavior of alkaloids on an RP column significantly decreases at low pH, and significantly increases at neutral pH 33, 34. To optimize the gradient and mass spectrometer conditions, analysis of a mixture of 18 authentic standards (Table 1) was performed with the UHPLC–QTOF system. To identify suitable chromatographic conditions, different elution systems were tested and their selectivity, sensitivity, resolution, tailing factor, and peak widths were considered as important parameters. First, a gradient system with the mobile phases of 5 mM ammonium formate in water (A) and acetonitrile (B) was optimized on C18 and pentafluorophenyl columns. Although the pentafluorophenyl column separated allocryptopine, protopine, and chelidonine well, which were not separated by the C18 column, it showed peak broadening of (dihydro)berberine and (dihydro)sanguinarine using 5 mM ammonium acetate or ammonium formate as mobile‐phase adducts. In addition, the repeatability was suboptimal because of retention time (RT) drift. The aqueous C18 column, compatible with 100% aqueous mobile phase, exhibited more reproducible RTs and was selected as the ideal analytical column for the LC–QTOF‐based metabolomic analysis of alkaloids in *P. rhoeas*. Then, three elution conditions were tested on the Acquity UPLC HSS T3 column; 5 mM ammonium formate, 0.1% formic acid, and a combination of 0.05% formic acid and 2.5 mM ammonium formate as additives. Based on the optimized gradient system, elutions with different additives were performed on the authentic standards and the results are shown in Figure 1. The chromatographic separation and resolution among authentic standards were good for the ammonium formate additive, as shown in Figure 1A, but peak tailing and broadening were observed. This resulted in peak splitting and difficulties during the metabolomics data pre‐processing for samples containing a complex mixture of metabolites. As shown in Figure 1B, the use of moderately acidic additives improved the chromatographic properties when compared to ammonium formate alone. The use of 0.05% formic acid together with the addition of 2.5 mM ammonium formate improved the chromatographic resolution and detection sensitivity of the alkaloids (Figure 1C), which agrees well with the literature 33.

### 3.3 Identification of the alkaloids

Eighteen authentic standards representing the major alkaloids in *Papaver* genus were analyzed by LC–QTOF with the column described in Section 2 and optimized LC conditions. Table 1 summarizes the chromatographic information (RTs) and MS‐based information (adduct forms, observed mass, product ions). Highly abundant protonated molecule [M+H]^+^ ions of most alkaloids, except for the peak no. 11 (berberine) and 14 (sanguinarine), were observed in the ESI mass spectra due to the strong basicity of the secondary or tertiary amine group. Characteristically, berberine and sanguinarine showed strong intensities of intact [M]^+^ ion in the ESI‐MS analysis because of the cation on the nitrogen atom as explained in the previous report 35.

**Figure 2:**
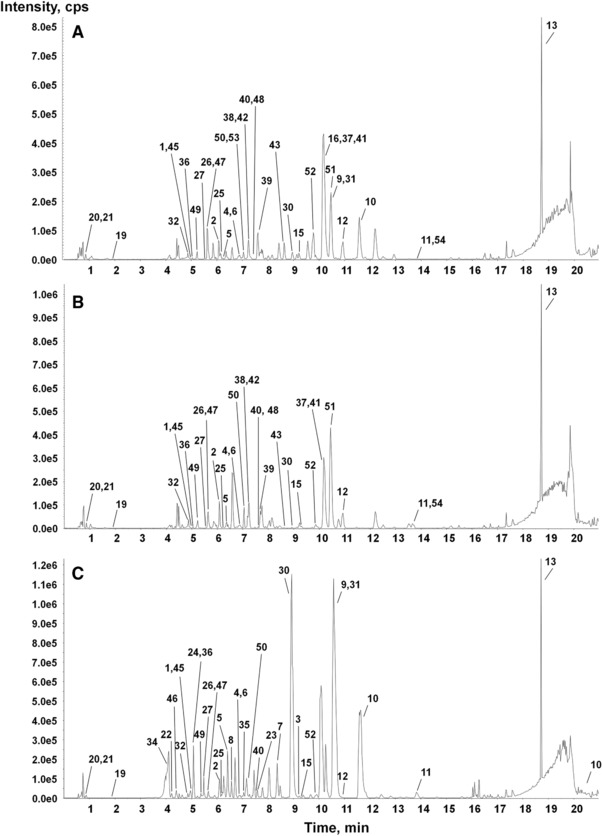
Representative base peak ion chromatograph (BPC) of the ethanol extracts of RS at 90 days (A), RA at 90 days (B), and PS at 90 days (C). The two types of *Papaver rhoeas* seeds have different origins; one (resource name: Shiri) originated from Moscow, Russia (RS) and the other (resource name: WS2981) originated from Hoengseong in the Province of Gangwon, South Korea (RA)

**Figure 3:**
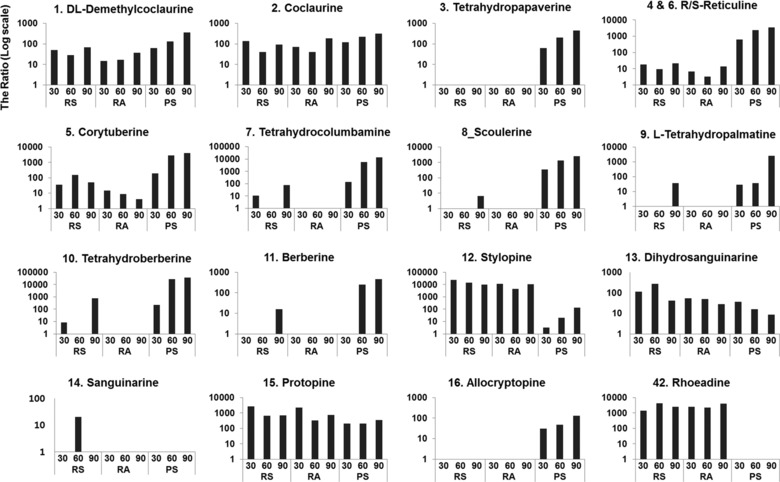
Representative metabolite intensities as determined by LC–QTOF analysis for samples at different growth stages (30, 60, and 90 cultivating days): dl‐demethylcoclaurine (1), coclaurine (2), tetrahydropapaverine (3), (*R*/*S*)‐reticuline (4 and 6), corytuberine (5), l‐tetrahydropalmatine (7), scoulerine (8), l‐tetrahydropalmatine (9), tetrahydroberberine (10), berberine (11), stylopine (12), dihydrosanguinarine (13), sanguinarine (14), protopine (15), allocryptopine (16), and rhoeadine (42). The ratio is the peak intensity of a metabolite in a sample that was divided by the peak intensity of a blank sample and expressed in a logarithmic scale. Compounds 1–16 were identified by authentic standards and the remaining 43 were identified tentatively by isotope patterns and comparison to database or previous literature

The MS/MS spectra of the authentic standards are shown in Supporting Information Figure S2 and were used for identification of alkaloids from the *P. rhoeas* (RS and RA) and *P. somniferum* (PS) samples. In addition, structural information such as formula and fragment patterns of many of the BIAs have been previously reported 2, 6, 8, 13, 14, 24, 30, 36. This information combined with the in‐house library on the Sciex instrument and online databases such as Metlin and MS bank were used for the tentative identification of various alkaloids in the RS, RA, and PS samples collected at 30, 60, and 90 days. As can be seen in Figure 2, the samples taken at 90 days show quite different chromatograms because they are composed of a larger variety of metabolites (the chromatograms of the other samples are shown in Supporting Information Figure S3). The identification of each component in the extract was first analyzed by comparison to authentic standards (Table 1) with MasterView in the PeakView software. Eight metabolites, demethylcoclaurine, coclaurine, (*R*/*S*)‐reticuline, stylopine, dihydrosanguinarine, and protopine were identified in the three samples during all growth stages even though their abundance varied depending on species and growth stage. Protopine (a protopine‐type alkaloid) and stylopine (a tetrahydroprotoberberine‐type alkaloid) from the RA and RS samples were more abundant during early growth stage (30 days) than at 60 and 90 days and could be characteristic metabolites of *P. rhoeas* (Figure 3). dl‐Demethylcoclaurine, coclaurine, and (*R*/*S*)‐reticuline (a benzylisoquinoline‐type alkaloid) and corytuberine (an aporphine‐type alkaloid) were observed in low abundance in RS and RA, but in the PS samples, these alkaloids significantly increased over the course of the growth process. Tetrahydrocolumbamine, tetrahydropalmatine, and tetrahydroberberine (a tetrahydroprotoberberine‐type alkaloid) and berberine (a protoberberine‐type alkaloid) were found in low abundance and only in the RS samples harvested at 30 and 90 days. Sanguinarine (a benzophenanthridine‐type alkaloid) was found only in the RS samples at 60 days. Also, dihyrosanguinarine (a benzophenanthridine‐type alkaloid) in the RS samples harvested at 60 days was significantly more abundant than the other specimens. Tetrahydropapaverine (a papaverine‐type alkaloid), scoulerine (a protoberberine‐type alkaloid), and allocryptopine (a protopine‐type alkaloid) were not observed in either RS or RA samples. Dihydroberberine (a protoberberine‐type alkaloid) and chelidonine (a benzophenanthridine‐type alkaloid) were not observed in any samples. According to the results, there is no significant change of representative metabolites in BIA biosynthesis from *P. rhoeas* (RS and RA).

For further identification of the alkaloids, the LC–QTOF base peak chromatogram collected in information‐dependent acquisition scan mode was processed to search and screen alkaloid metabolites by exact masses against an in‐house library and databases such as Chemspider, Metlin, and MS bank. The proposed identity of the alkaloid was compared to the RT and characteristic product ions (fragmentation patterns) in the reference MS/MS spectra. A total of 54 alkaloids were identified or tentatively identified in the base peak chromatograms of the RA, RS, and PS samples. Table 2 lists the benzylisoquinoline alkaloids that could be authentically or tentatively identified in the samples by the developed LC–QTOF‐MS/MS method. Alkaloids identified or tentatively identified in the aerial parts of the cultivated *P. rhoeas* and *P. somniferum* are summarized in Supporting Information Table S2. Also, metabolites (colored or bold text) from *P. rhoeas* (RS and RA) identified in benzylisoquinoline alkaloid (BIA) biosynthesis pathways 24 were marked as shown in Supporting Information Figure S4. Further analysis of precursors of BIA was conducted by LC–QTOF. The precursors such as tyrosine, 4‐hydroxyphenyacetaldehyde, tyramine, dopamine due to polarity were effectively extracted with distilled water from aerial parts of RS and RA. Those were identified, and intensities as determined by LC–QTOF analysis for samples at different growth stages (30, 60, and 90 cultivating days) are expressed in Supporting Information Figure S4. Tyramine, dopamine, and 4‐hydroxyphenyacetaldehyde increased during growth, and the contents of tyramine increased significantly. Stylopine, protopine, and rhoeadine were mainly observed as secondary metabolites over all stages from the aerial parts of *P. rhoeas*.

**Table 2:** Characterization of the benzylisoquinoline alkaloids by LC–QTOF‐MS

**Peak no**.	**Name**	**Formula**	**Expected RT (min)**	**Predicted mass (Da)**	**Adduct form [M]^+^ or [M+H]^+^**	**Observed mass (Da)**	**Product ions (MS/MS fragment ions, *m/z*)**
1	DL‐Demethylcoclaurine	C_16_H_17_NO_3_	4.98	271.1208	[M+H]^+^	272.1281	107.0493, 255.1015, 161.0591, 143.0490
2	Coclaurine	C_17_H_19_NO_3_	6.03	285.1365	[M+H]^+^	286.1438	107.0494, 269.1167, 175.0744, 237.0905
3	Tetrahydropapaverine	C_20_H_25_NO_4_	9.11	343.1784	[M+H]^+^	344.1856	192.1023, 189.0912, 151.0757, 327.1600
4 & 6	Reticuline	C_19_H_23_NO_4_	6.74	329.1627	[M+H]^+^	330.1700	192.1017, 137.0598, 143.0490, 175.0749
5	Corytuberine	C_19_H_21_NO_4_	6.28	327.1471	[M+H]^+^	328.1543	265.0859, 237.0908, 297.1122, 205.0644
7	Tetrahydrocolumbamine	C_20_H_23_NO_4_	8.38	341.1627	[M+H]^+^	342.1700	178.0870, 163.0634, 176.0711
8	Scoulerine	C_19_H_21_NO_4_	6.57	327.1471	[M+H]^+^	328.1543	237.0919, 207.0445, 211.0758, 239.0707
9	l‐Tetrahydropalmatine	C_21_H_25_NO_4_	10.53	355.1784	[M+H]^+^	356.1856	192.1017, 165.0905, 176.0700
10	Tetrahydroberberine (canadine)	C_20_H_21_NO_4_	11.68	339.1471	[M+H]^+^	340.1543	176.0707, 149.0595, 174.0548
11	Berberine	C_20_H_18_NO_4_	13.83	336.1236	[M]^+^	336.1236	320.0922, 292.0970, 321.1000, 306.0762, 278.0813
12	Stylopine	C_19_H_17_NO_4_	10.94	323.1158	[M+H]^+^	324.1230	176.0705, 149.0596
13	Dihydrosanguinarine	C_20_H_15_NO_4_	19.10	333.1001	[M+H]^+^	334.1074	318.0764, 319.0842, 304.0967, 276.1016
14	Sanguinarine	C_20_H_14_NO_4_	12.77	332.0923	[M]^+^	332.0923	317.0683, 274.0861, 304.0969
15	Protopine	C_20_H_19_NO_5_	9.22	353.1263	[M+H]^+^	354.1336	188.0702, 189.0778, 149.0591
16	Allocryptopine	C_21_H_23_NO_5_	10.21	369.1576	[M+H]^+^	370.1649	188.0701, 189.0780, 290.0939
17	Chelidonine	C_20_H_19_NO_5_	10.18	353.1263	[M+H]^+^	354.1336	275.0704, 247.852, 305.0815, 323.0917
18	Dihydroberberine	C_20_H_19_NO_4_	10.95	337.1314	[M+H]^+^	338.1387	322.1071, 294.1123, 308.0914, 279.0876
19	Tyramine	C_8_H_11_NO	1.91	137.0841	[M+H]^+^	138.0913	77.0401, 121.0649, 103.0536, 91.0551
20	Dopamine	C_8_H_11_NO_2_	0.89	153.0790	[M+H]^+^	154.0863	91.0599, 137.0600, 119.0496
21	4‐Hydroxyphenylacetaldehyde	C_8_H_8_NO_2_	0.89	136.0524	[M+H]^+^	137.0597	91.0551, 65.0420, 119.0501, 63.0269
22	Morphine	C_17_H_19_NO_3_	4.10	285.1365	[M+H]^+^	286.1438	201.0900, 229.0849, 185.0584, 211.0747
23	Mecambrine	C_18_H_17_NO_3_	7.68	295.1208	[M+H]^+^	296.1281	202.0853, 171.0674, 280.0965
24	Codeine	C_18_H_21_NO_3_	5.08	299.1521	[M+H]^+^	300.1594	215.1064, 243.1013, 225.0906, 199.0748
25	(*S*)‐*N‐*Methylcoclaurine	C_18_H_21_NO_3_	6.09	299.1521	[M+H]^+^	300.1594	269.1180, 107.0495, 271.1348
26	Armepavine	C_19_H_23_NO_3_	5.64	313.1678	[M+H]^+^	314.1751	107.0495, 58.0675, 269.1160, 271.1328, 298.1070
27	(*S*)‐3′‐Hydroxy‐*N*‐methylcoclaurine	C_18_H_21_NO_4_	5.53	315.1471	[M+H]^+^	316.1543	192.1010, 123.0430, 285.1116, 300.1196
28	(*S*)‐Cheilanthifoline	C_19_H_19_NO_4_	7.70	325.1314	[M+H]^+^	326.1387	178.0865, 190.0862, 163.0625
29	Papaverine	C_20_H_21_NO_4_	10.15	339.1471	[M+H]^+^	340.1543	202.0867, 324.1216, 296.1288, 171.0683
30	Cryptopine	C_21_H_23_NO_5_	8.97	369.1576	[M+H]^+^	370.1649	352.1190, 205.1099, 165.0913, 190.0862
31	Noscapine	C_22_H_23_NO_7_	10.59	413.1475	[M+H]^+^	414.1547	220.0967, 353.1030, 365.1032, 179.0705
32	4‐Hydroxyphenylpyruvate	C_9_H_8_O_4_	4.86	180.0423	[M+H]^+^	181.0495	89.0402, 135.0438, 163.0396, 145.0291, 117.0345
33	Codeinone	C_18_H_19_NO_3_	5.64	297.1365	[M+H]^+^	298.1438	283.1208, 282.1125, 254.1180, 266.1213
34	Morphine *N*‐oxide	C_17_H_19_NO_4_	4.16	301.1314	[M+H]^+^	302.1387	284.1280, 241.0860
35	Flavinantine	C_19_H_21_NO_4_	7.17	327.1471	[M+H]^+^	328.1543	178.0855, 163.0621
36	8,14‐dihydroflavinantine (or salutaridinol)	C_19_H_23_NO_4_	5.05	329.1627	[M+H]^+^	330.1700	285.1121, 123.0435, 58.0665, 143.0482
37	(*S*)‐*cis*‐*N*‐Methylstylopine	C_20_H_20_NO_4_	10.20	338.1392	[M+H]^+^	339.1465	191.0891, 190.0857, 149.0589
38	Isocorydine	C_20_H_23_NO_4_	7.25	341.1627	[M+H]^+^	342.1700	297.1120, 265.0856, 237.0904
39	Pseudoprotopine	C_20_H_19_NO_5_	7.69	353.1263	[M+H]^+^	354.1336	188.0703, 189.0775, 149.0592
40	Amurensinine *N*‐oxide A (or amurensinine *N*‐oxide B)	C_20_H_21_NO_5_	7.61	355.1420	[M+H]^+^	356.1493	190.0567, 191.0944, 277.0864, 151.0756
41	Rheagenine (or isorheagenine)	C_20_H_19_NO_6_	10.19	369.1212	[M+H]^+^	370.1285	352.1185, 340.1180, 324.1226
42	Rhoeadine (or isorhoeadine)	C_21_H_21_NO_6_	7.27	383.1369	[M+H]^+^	384.1442	321.0763, 303.0649, 291.0653, 366.1341
43	Glaucamine (or isoglaucamine)	C_21_H_23_NO_6c_	8.64	385.1525	[M+H]^+^	386.1598	368.1499, 338.1042
44	Coptisine	C_19_H_14_NO_4_	10.75	320.0923	[M]^+^	320.0923	292.0964, 277.0728, 290.0805, 318.0755, 262.0858
45	Unknown M1	C_16_H_17_NO_3_	4.98	271.1208	[M+H]^+^	272.1281	227.1772, 107.0496, 161.0580, 209.1688, 255.1030
46	Unknown M2	C_17_H_17_NO_3_	4.32	283.1208	[M+H]^+^	284.1281	175.0568, 129.0169, 203.0549, 227.0707
47	Unknown M3	C_18_H_19_NO_3_	5.64	297.1365	[M+H]^+^	298.1438	283.1211, 254.1180, 177.0774, 148.0776, 107.0498
48	Unknown M4	C_19_H_21_NO_3_	7.60	311.1521	[M+H]^+^	312.1594	267.1008, 252.0774, 181.0599, 121.0299
49	Unknown M5	C_18_H_21_NO_4_	5.32	315.1471	[M+H]^+^	316.1543	298.1430, 283.1218, 254.1169, 121.0647
50	Unknown M6	C_19_H_21_NO_4_	7.18	327.1471	[M+H]^+^	328.1543	178.0855, 163.0616, 151.0750, 91.0572, 176.0685
51	Unknown M7	C_21_H_21_NO_6_	10.45	383.1369	[M+H]^+^	384.1442	352.1180, 190.0863, 188.0710, 303.0653, 334.1077
52	Unknown M8	C_21_H_24_NO_4_	9.81	354.1705	[M+H]^+^	355.1778	207.1204, 206.1172, 191.0913,
53	Unknown M9	C_22_H_25_NO_6_	7.22	399.1682	[M+H]^+^	400.1755	337.1073, 319.0962, 204.1006, 58.0684, 382.1637
54	Unknown M10	C_20_H_19_NO_4_	13.81	337.1314	[M+H]^+^	338.1387	321.0953, 322.1021, 293.1003, 307.0820
55	Unknown M11	C_21_H_21_NO_6_	12.20	383.1369	[M+H]^+^	384.1442	352.1180, 190.0861, 320.0923, 291.0654, 263.0704

Peak no. 1–18 identified by direct comparison to authentic standards.

Peak no. 19 and 20 tentatively identified by comparison to the in‐house MS/MS library (Sciex) and Metlin database.

Peak no. 21–29 tentatively identified by comparison to the Metlin database.

Peak no. 30 and 31 tentatively identified by comparison to the MS bank database.

Peak no. 32–44 tentatively identified by isotope MS pattern and previous reports.

Peak no. 45–55 unknown.

## 4 CONCLUDING REMARKS

In this study, an effective LC–QTOF‐MS/MS method was developed for the simultaneous identification of alkaloids in *Papaver* species. Alkaloids in *P. rhoeas* were separated and analyzed by LC–QTOF in a single run. Simultaneously, narcotic substances such as morphine and codeine derived from the opium poppy could be monitored. This could be helpful in the discrimination between species in the genus *Papaver*, as some are controlled substances whereas others are commonly found ornamental plants. In this first attempt to investigate the content, abundance, and identity of alkaloids present during the growth of *P. rhoeas*, information related to the growth stage and origin could be useful in understanding the biosynthesis and metabolism of alkaloids in *P. rhoeas*, even those in low abundance using MS‐based metabolite profiling.

## CONFLICT OF INTEREST

The authors have declared no conflict of interest.

## Supporting information

Supporting Information
