# Supplementary material for: Identification and metabolite profiling of alkaloids in aerial parts of Papaver rhoeas by liquid chromatography coupled with quadrupole time‐of‐flight tandem mass spectrometry
Source: J Sep Sci. 2018 Apr 25;41(12):2517–27. doi: 10.1002/jssc.201701402 (PMC6032884; doi:10.1002/jssc.201701402)
Supplement: Supplementary file 1 — Supporting Information [file JSSC-41-2517-s001.docx]

**Supporting Information**

**Identification and metabolite profiling of alkaloids in aerial parts of *Papaver rhoeas* by liquid chromatography coupled with quadrupole time-of-flight tandem mass spectrometry**

Jae-Hyeon Oh^1†^, In Jin Ha^2†^, Min Young Lee^2^, Eun-Ok Kim^2^, Dain Park^2^, Jun-Hee Lee^2,4^, Seok-Geun Lee^2,3^, Do-Wan Kim^1^, Tae-Ho Lee^1^, Eui-Ju Lee^2,4^*, Chang-Kug Kim^1^*

^1^ Genomics Division, Department of Agricultural biotechnology, National Institute of Agricultural Science (NAS), Rural Development Administration (RDA), Jeollabuk-do 54874, Republic of Korea

^2^ Korean Medicine Clinical Trial Center (K-CTC), Kyung Hee University Korean Medicine Hospital, Seoul 02447, Republic of Korea

^3^ KHU-KIST Department of Converging Science & Technology, Kyung Hee University, Seoul 02447, Republic of Korea

^4^ Department of Sasang Constitutional Medicine, College of Korean Medicine, Kyung Hee University, Seoul 02447, Republic of Korea

^†^ In Jin Ha and Jae-Hyeon Oh made an equal contribution to this work.

**List of supporting information**

**Table S1**. Peak areas of precursor-product ion pairs of protopine analyzed by LC-QTRAP (p. 3)

**Figure S1**. Multiple Reaction Monitoring (MRM) chromatograms of (A) the authentic standard of protopine and (B) samples extracted with difference solvents in positive

**Figure S2**. QTOF MS/MS spectra of [M+H]+ or [M]+ ions of authentic standards (a) DL-Demethylcoclaurine, (b) Coclaurine, (c) Tetrahydropapaverine, (d) R/S-Reticuline, (e) Corytuberine, (f) L-Tetrahydropalmatine, (g) Scoulerine, (h) L-Tetrahydropalmatine, (i) Tetrahydroberberine, (j) Berberine, (k) Stylopine, (l) Dihydrosanguinarine, (m) Sanguinarine, (n) Protopine, (o) Allocryptopine, (p) Chelidonine, (q) Dihydroberberine. (p. 4-9)

**Figure S3**. Base peak ion chromatogram (BPC) of samples: (a) RS at a cultivation period of 30 days, (b) RA at 30 days, (c) PS at 30 days, (d) RS at 60 days, (e) RA at 60days, and (f) PS at 60 days (p. 10-12)

**Figure S4.** Metabolites (colored or bold text) from *Papaver rhoeas* (RS and RA) identified in benylisoquinoline alkaloid (BIA) biosynthesis pathways

**Table S2**. Identification of alkaloids authentically or tentatively by analyzed by LC-QTOF in the ethanol extracts of aerial parts of *P. rhoeas* and *P. somniferum*. at different growing stages (p. 13-15)

**Table S1.** Peak areas of precursor-product ion pairs of protopine analyzed by LC-QTRAP

| **Extraction solvents** | **354.0/ 188.2** | **354.0/ 189.2** | **354.0/ 149.1** | **354.0/ 91.1** |
| --- | --- | --- | --- | --- |
| 50% Methanol | (5) 124000 | (5) 131300 | (5) 61337 | (5) 29733 |
| 80% Methanol | (3) 144300 | (3) 151367 | (3) 75333 | (3) 34160 |
| 100% Methanol | (4) 140067 | (4 149933 | (4) 68673 | (4) 32503 |
| 100% Ethanol | (2) 177333 | (2) 185300 | (2) 83740 | (2) 39467 |
| Ethylether + 10% ammonia | (1) 211933 | (1) 223433 | (1) 102073 | (1) 49510 |

* (number): ranks

**Figure S1**. Multiple Reaction Monitoring (MRM) chromatograms of (A) the authentic standard of protopine and (B) samples extracted with difference solvents in positive

**
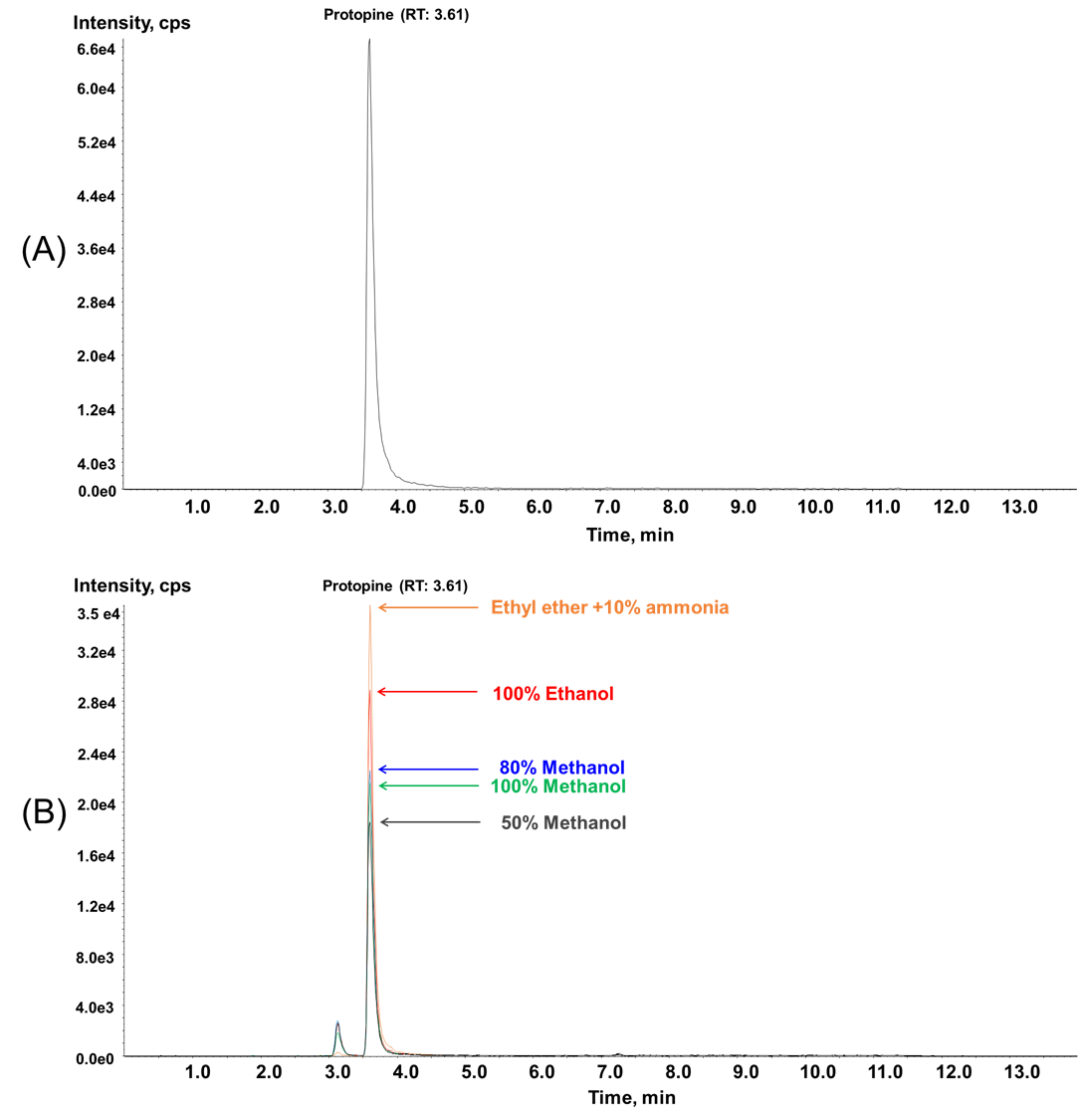
**

**Figure S2**. QTOF MS/MS spectra of [M+H]+ or [M]+ ions of authentic standards (a) DL-Demethylcoclaurine, (b) Coclaurine, (c) Tetrahydropapaverine, (d) R/S-Reticuline, (e) Corytuberine, (f) L-Tetrahydropalmatine, (g) Scoulerine, (h) L-Tetrahydropalmatine, (i) Tetrahydroberberine, (j) Berberine, (k) Stylopine, (l) Dihydrosanguinarine, (m) Sanguinarine, (n) Protopine, (o) Allocryptopine, (p) Chelidonine, (q) Dihydroberberine

**
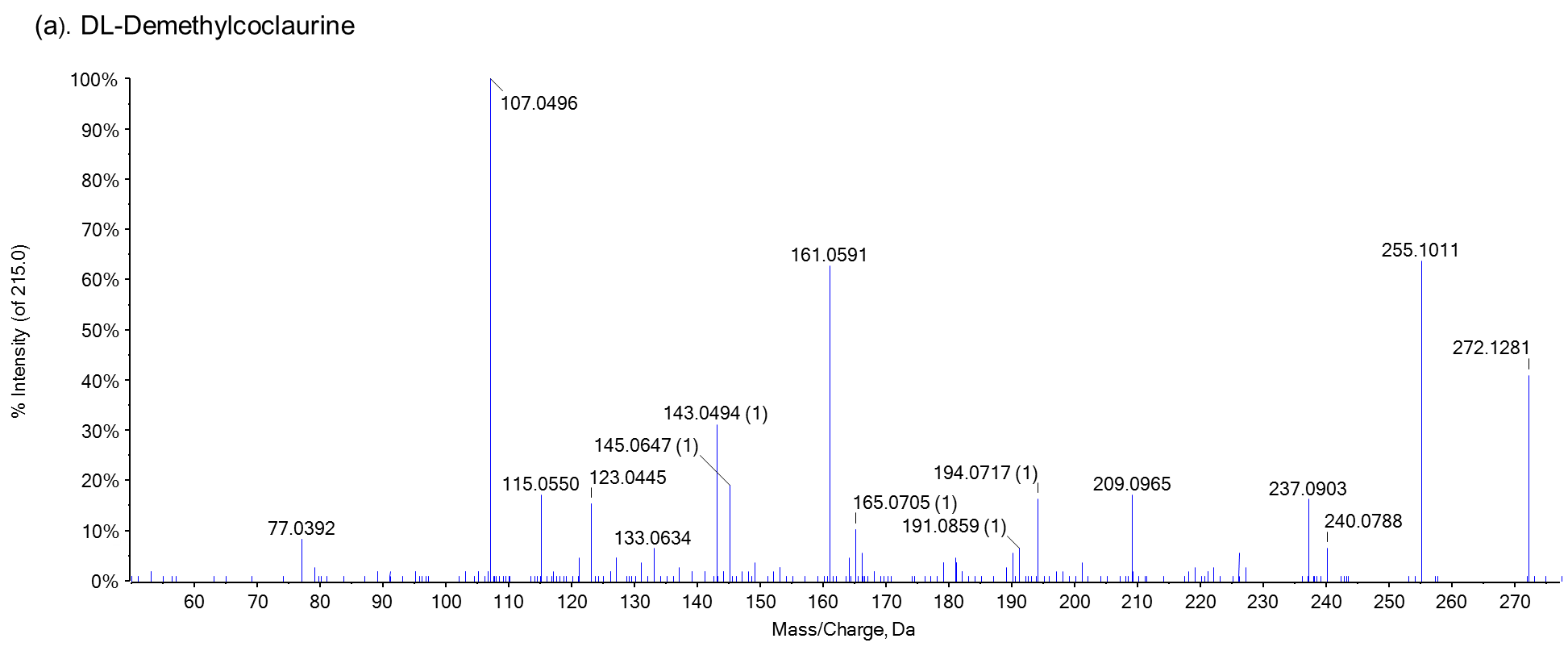
** **
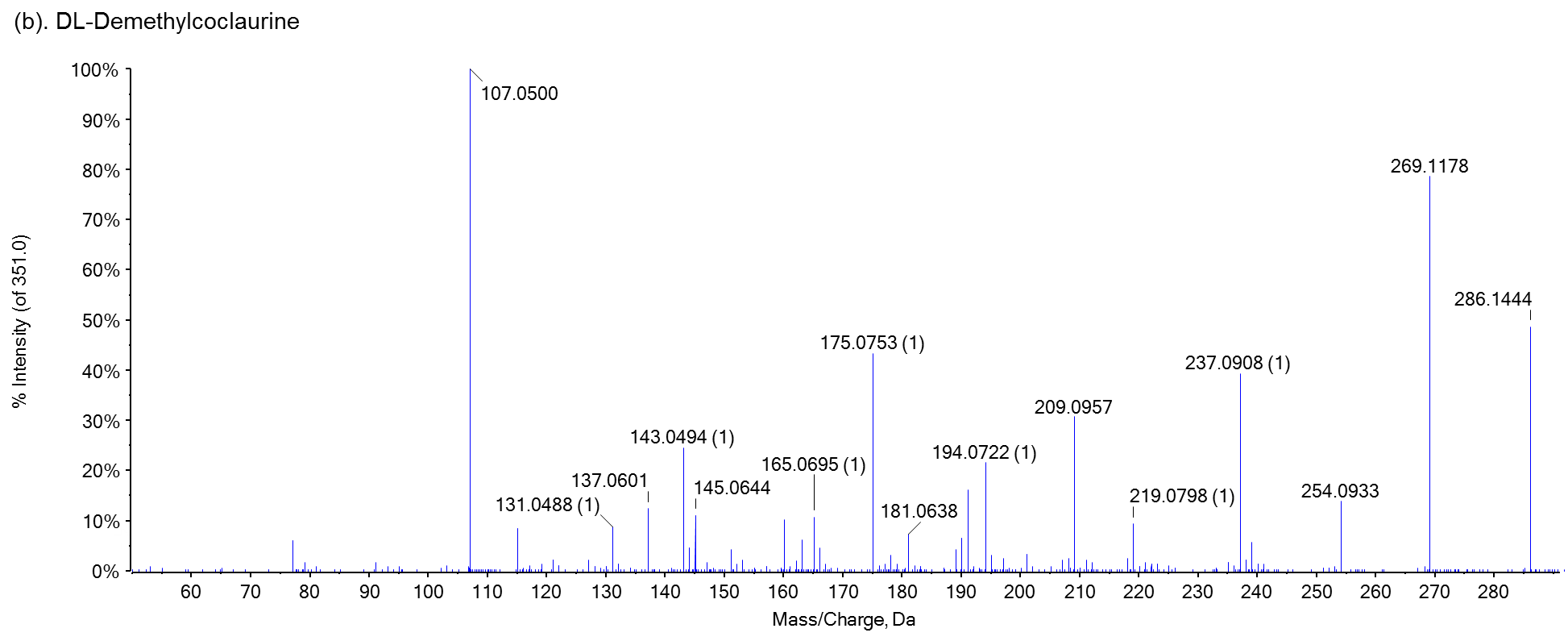
**

**
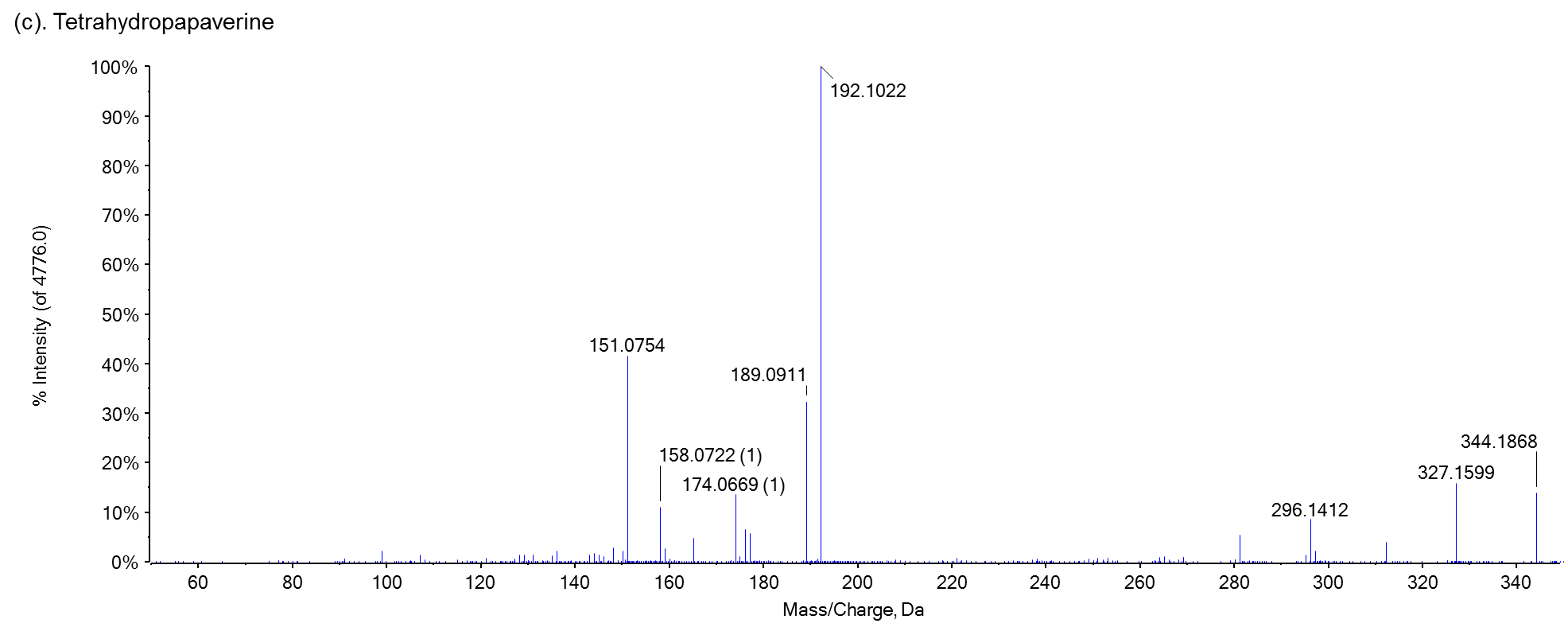
**

**
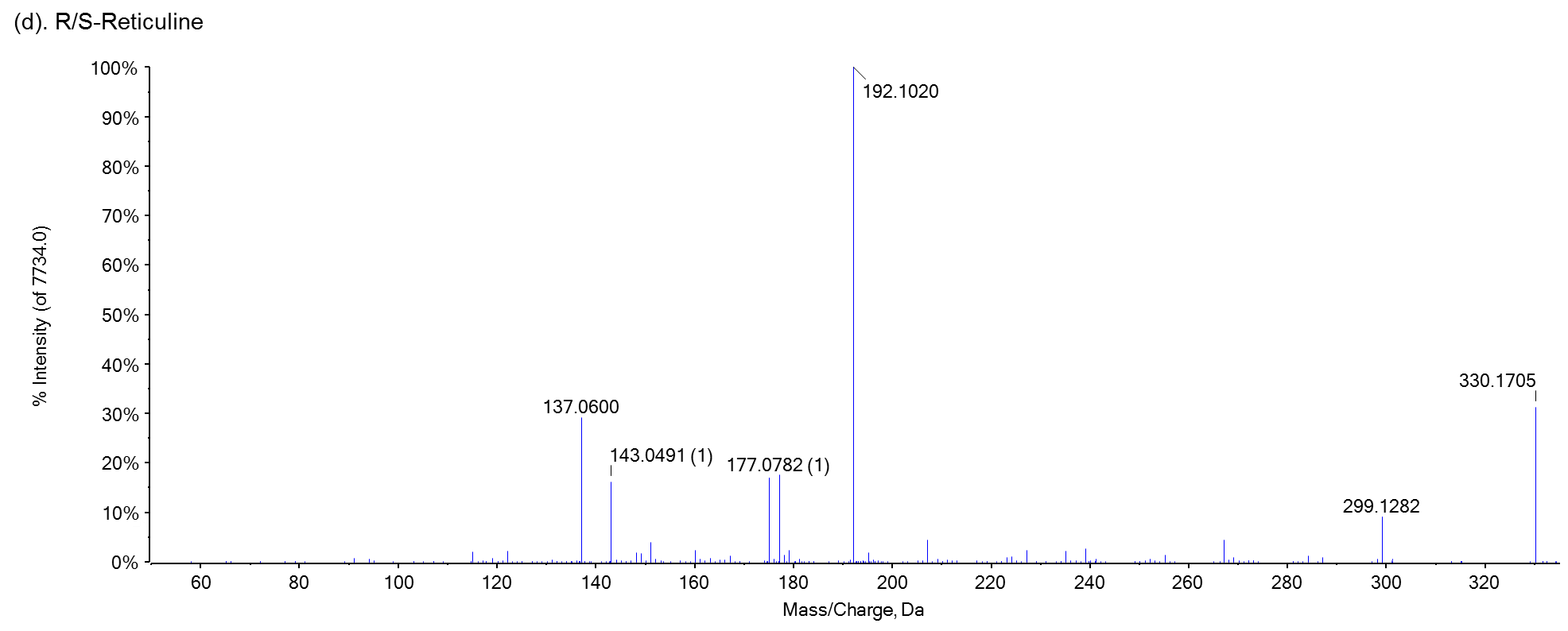
** **
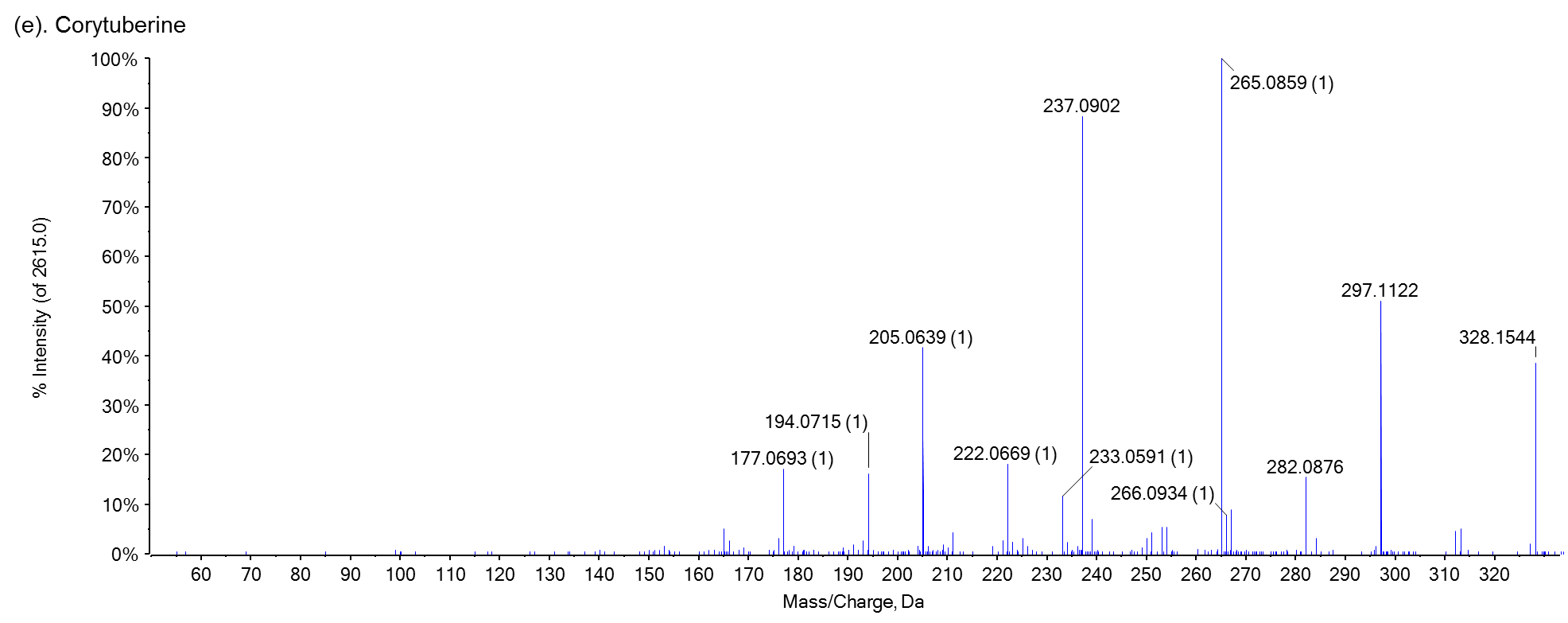
**
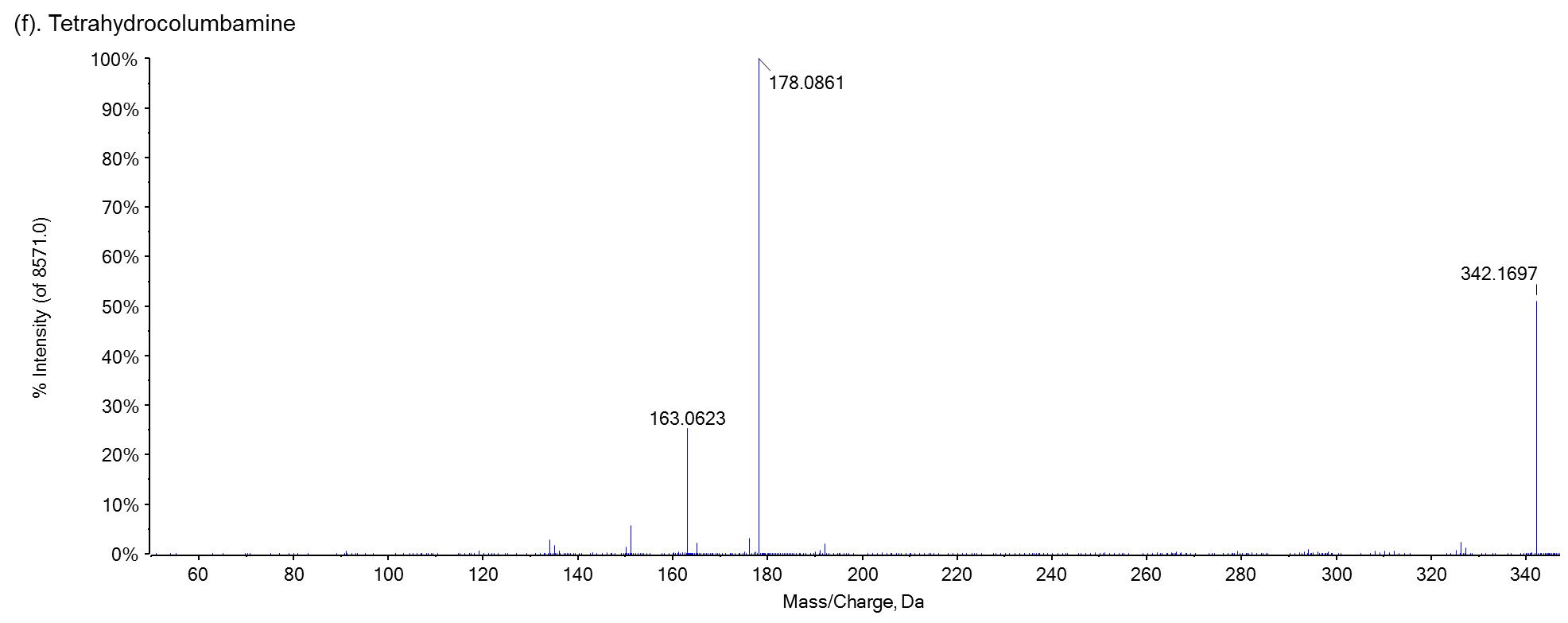


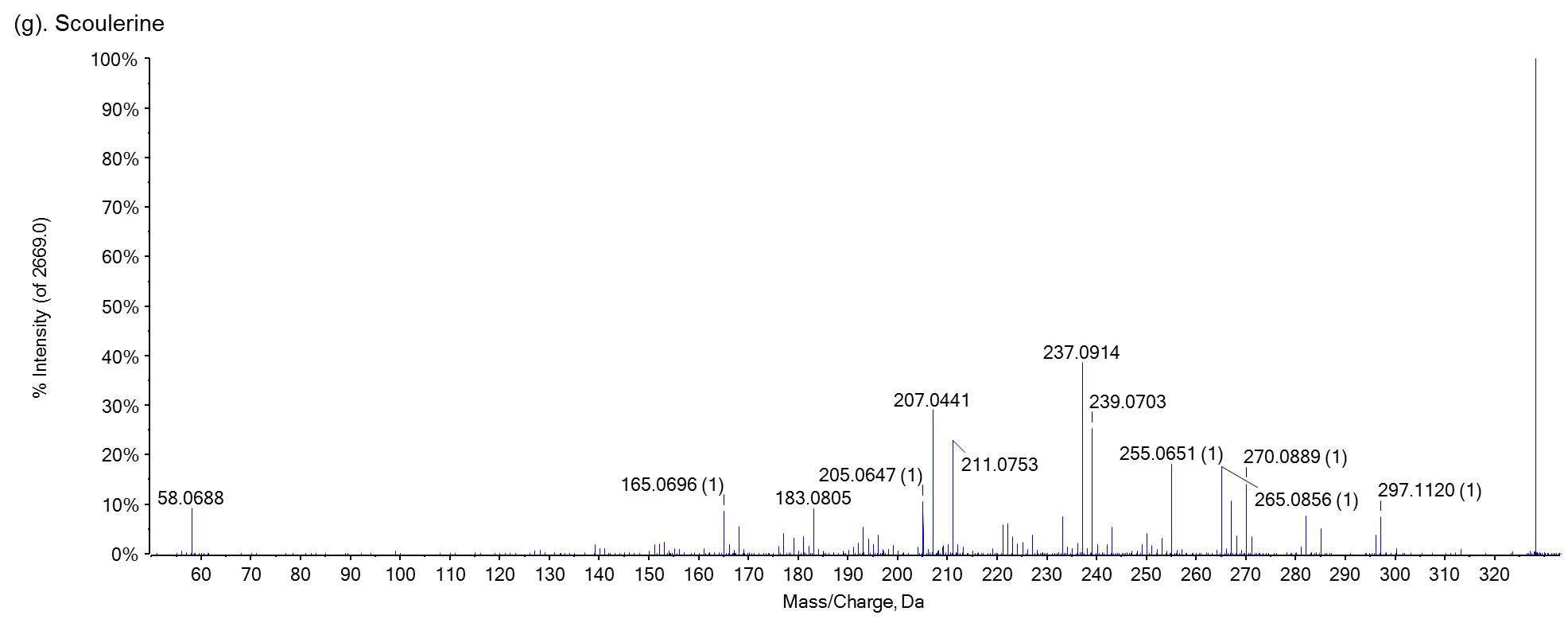


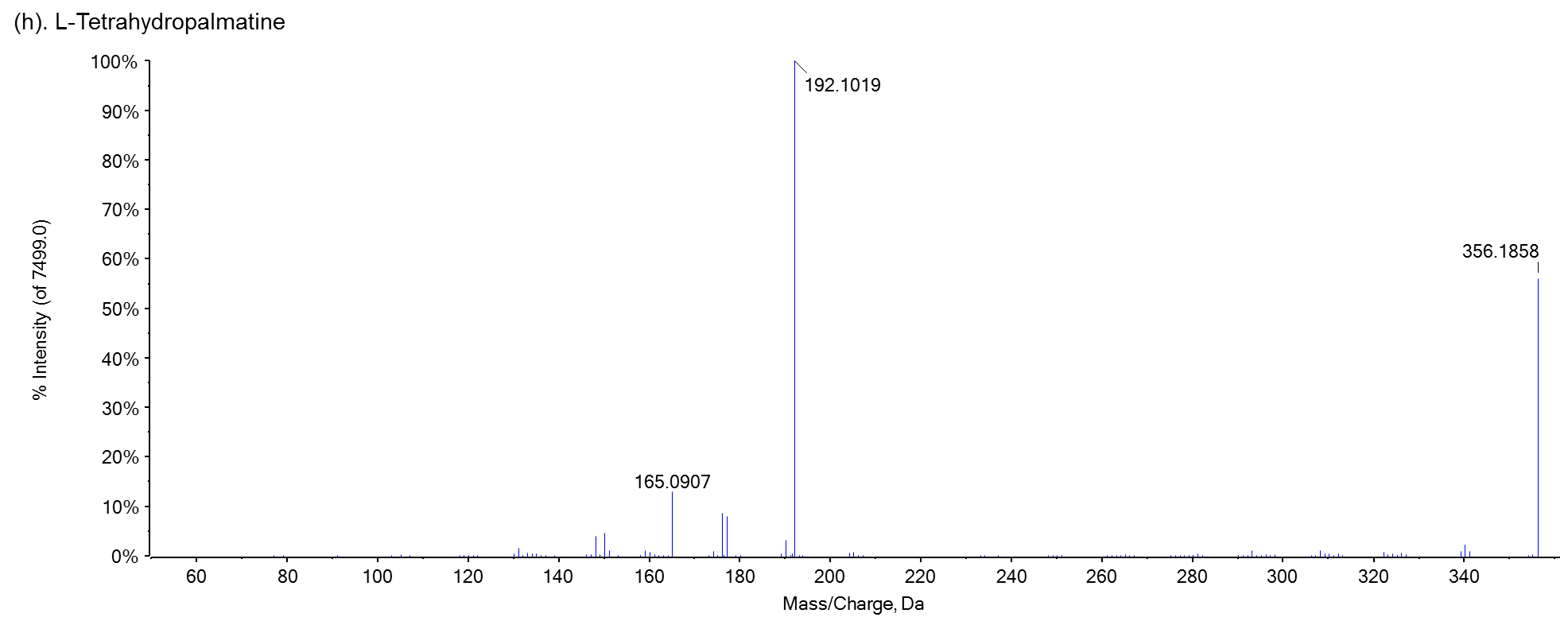


**
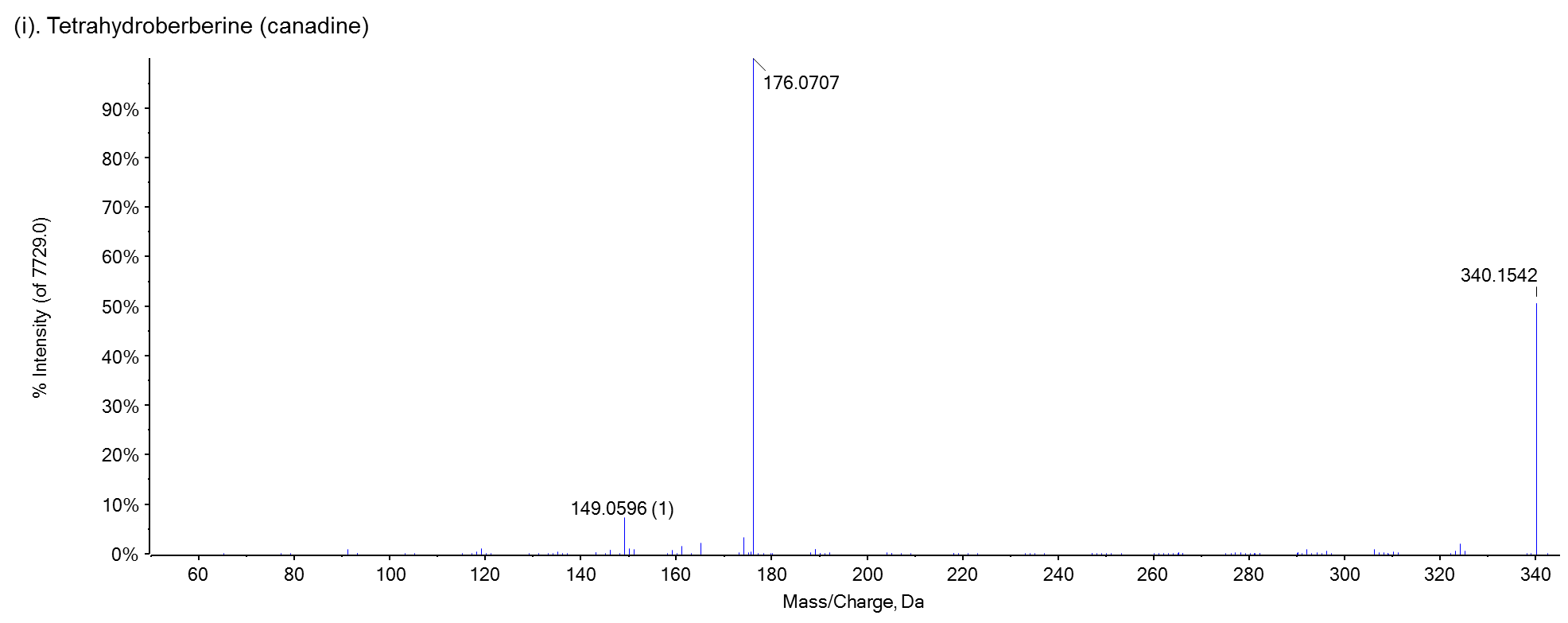
**

**
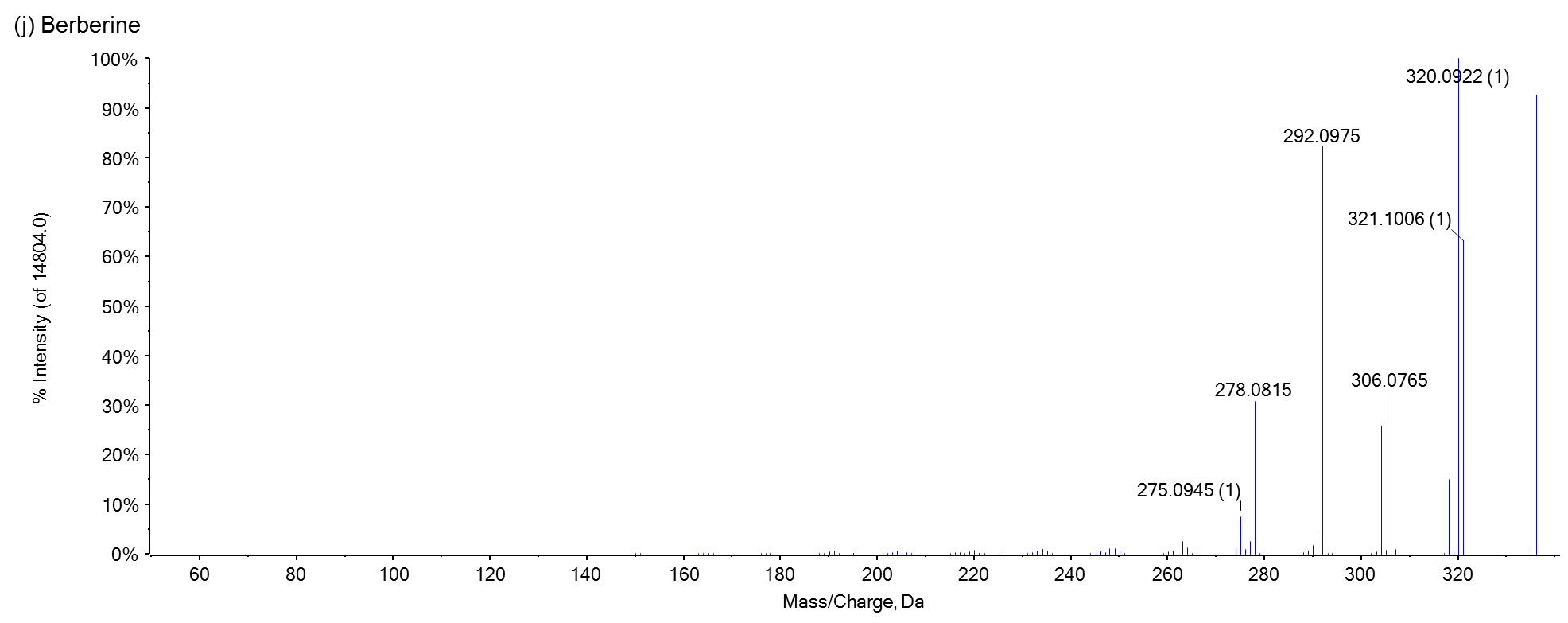
** **
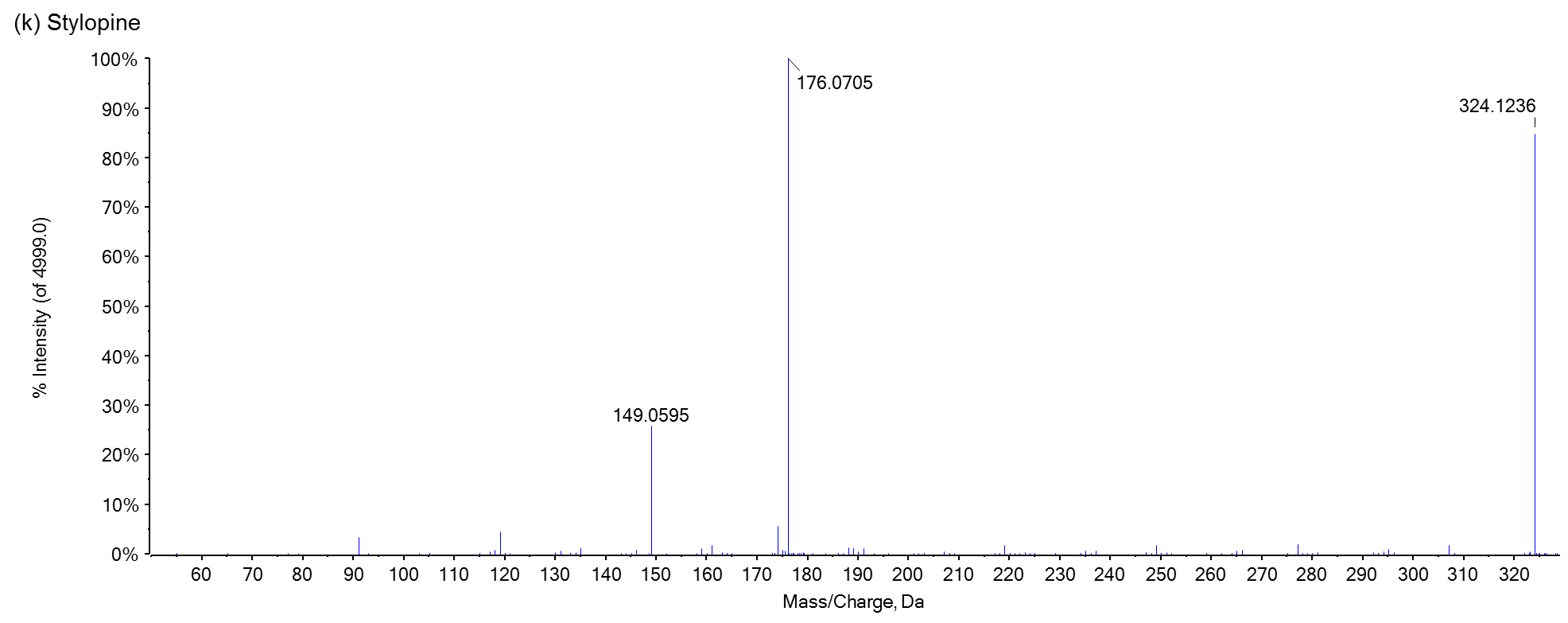
**
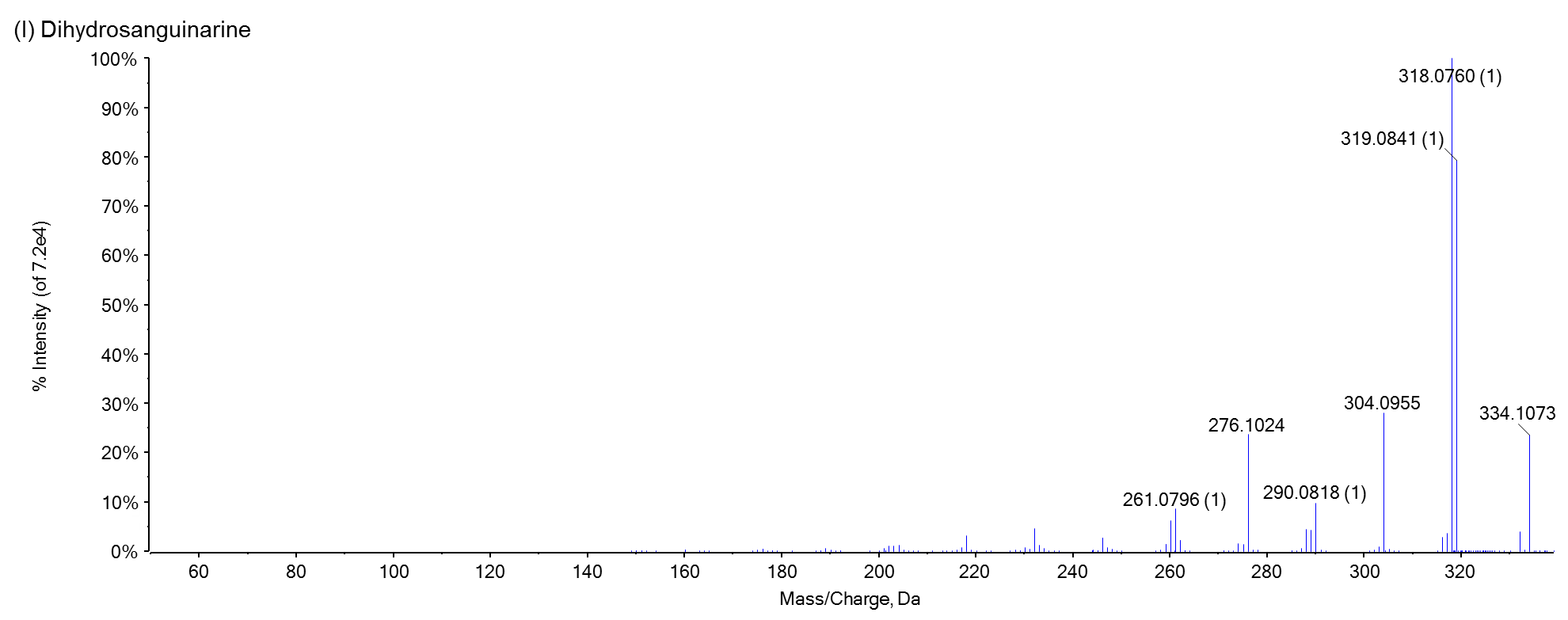


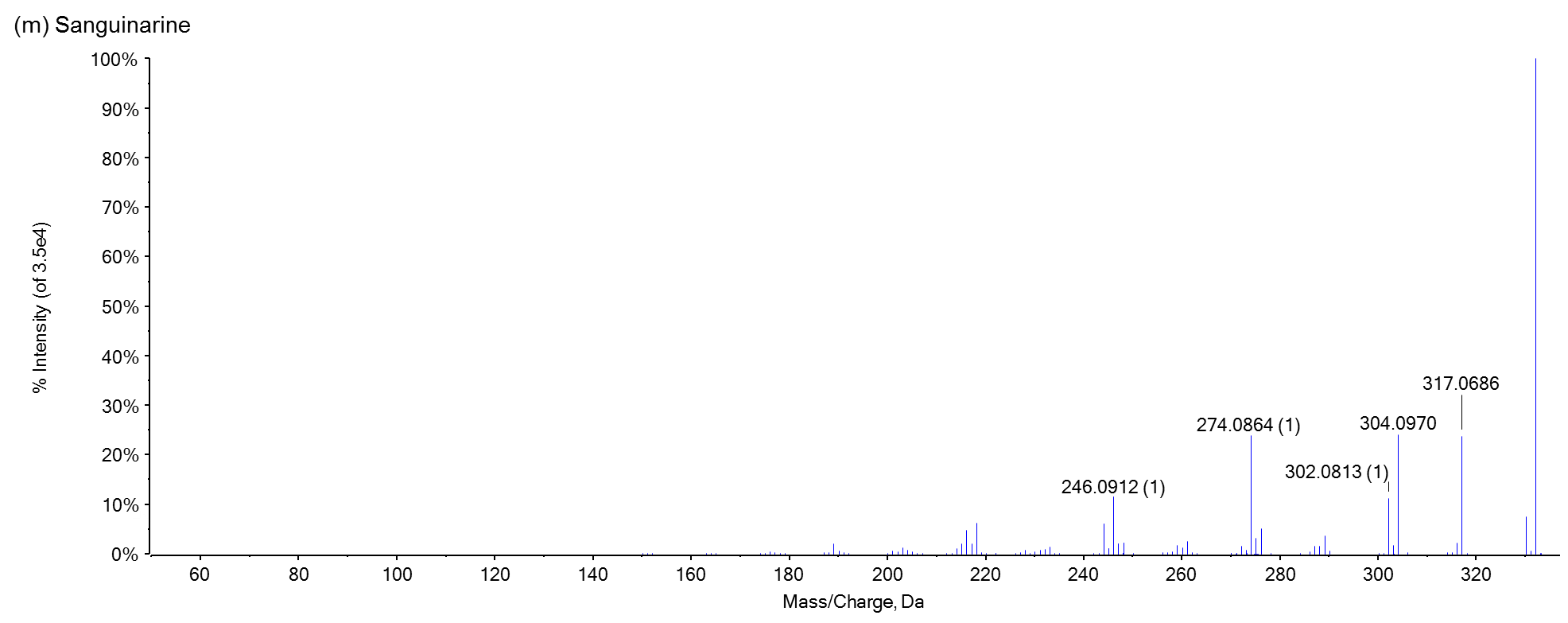


**
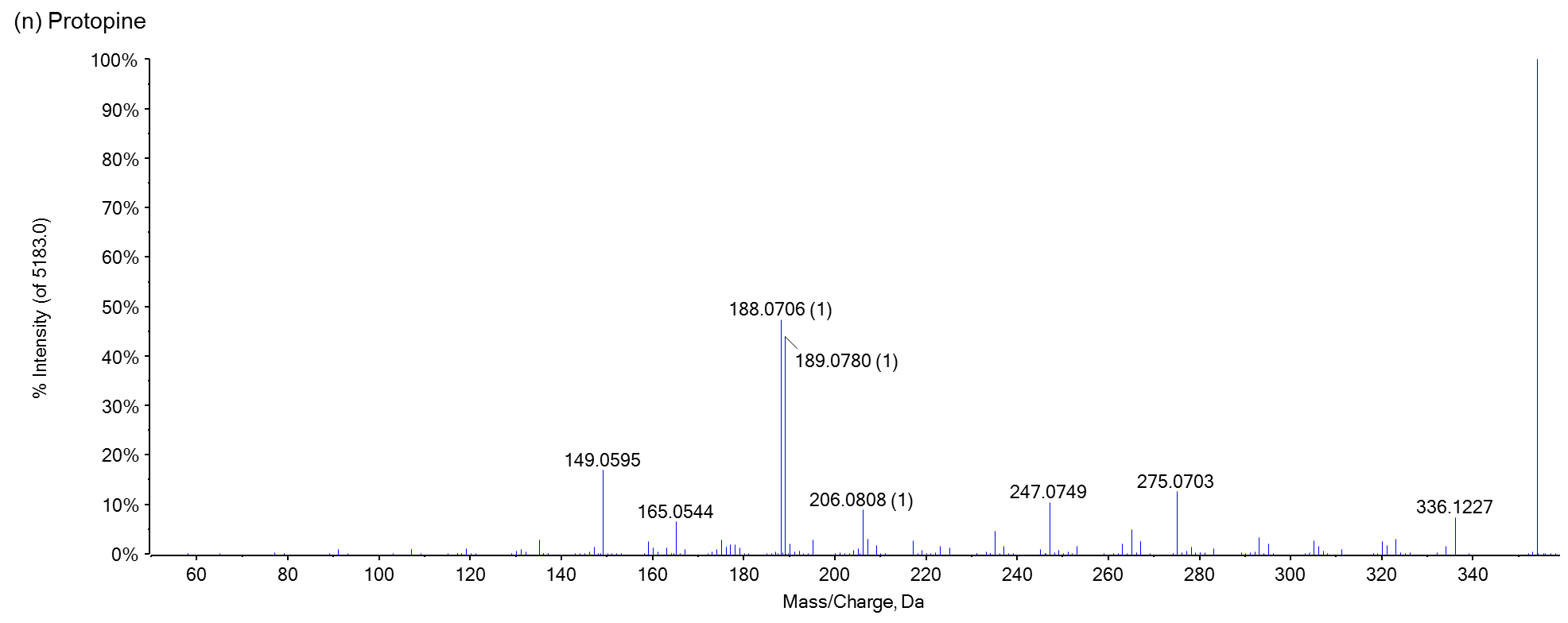
**

**
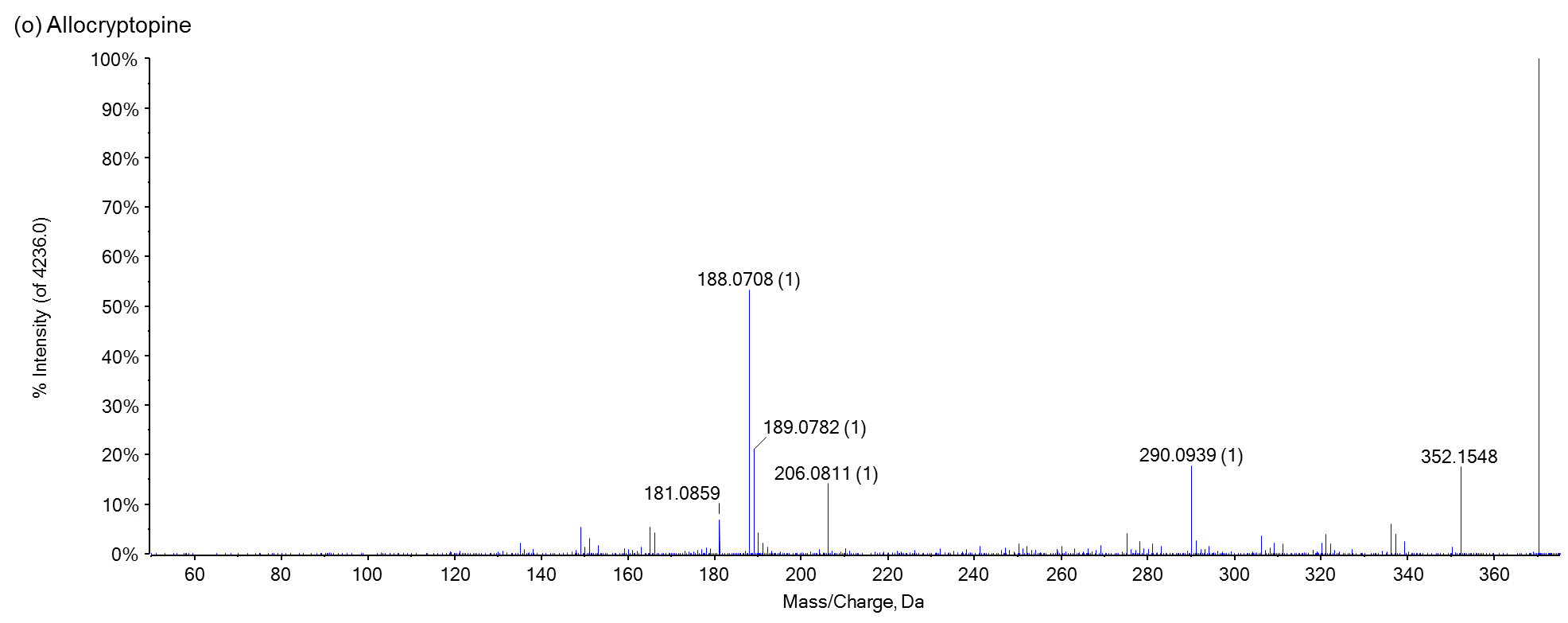
**

**
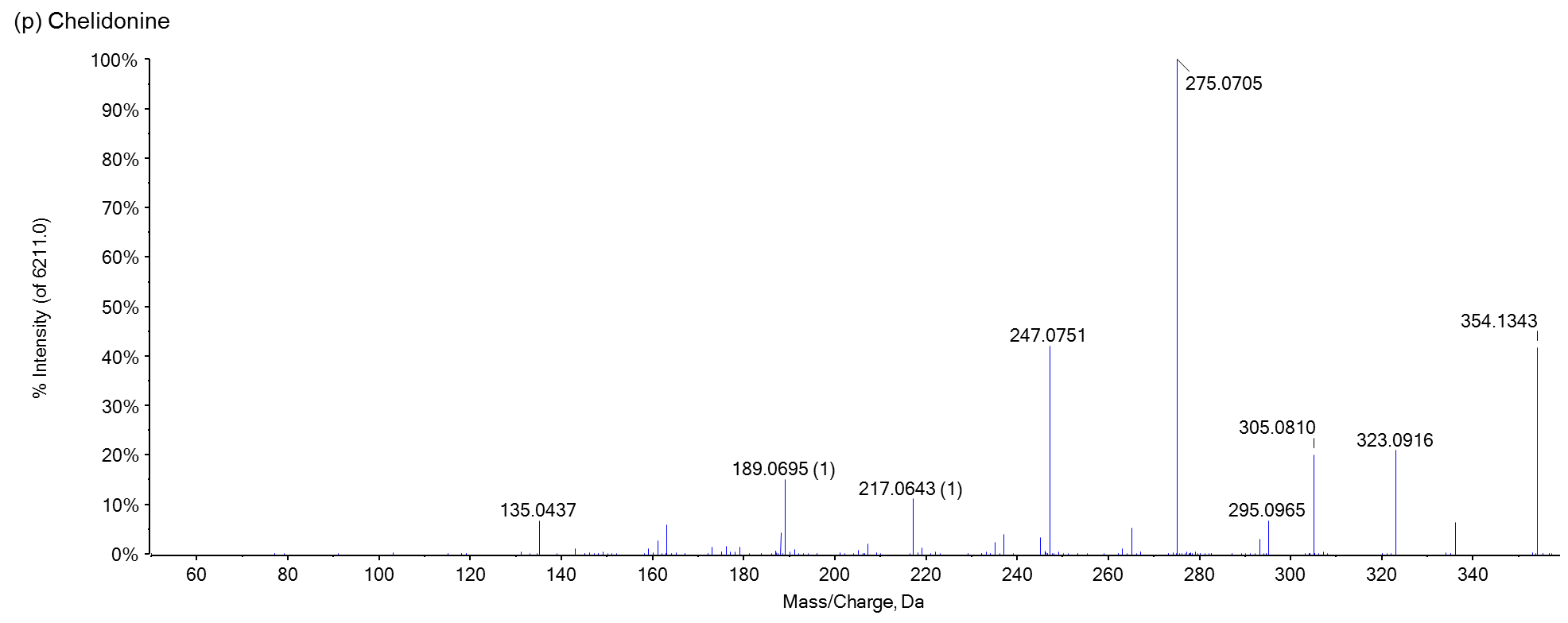
**

**
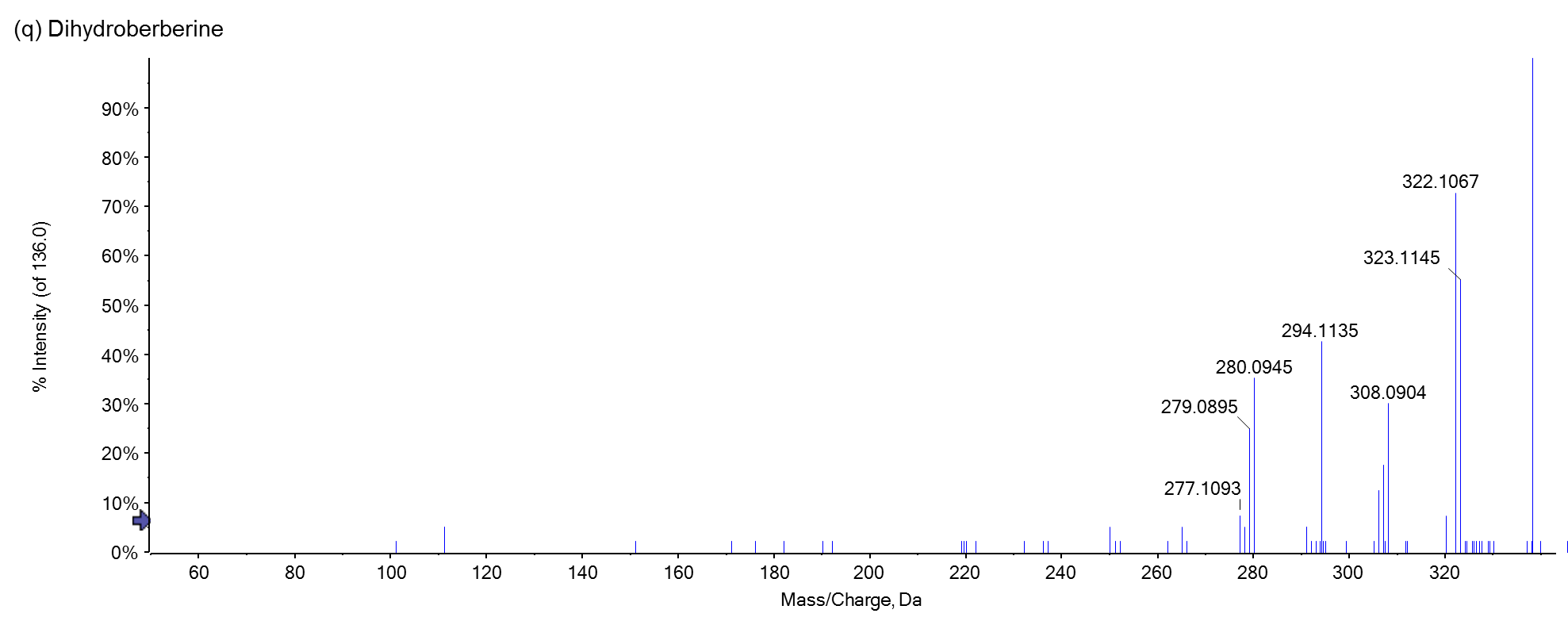
**

**Figure S3**. Base peak ion chromatogram (BPC) of samples: (a) RS at a cultivation period of 30 days, (b) RA at 30 days, (c) PS at 30 days, (d) RS at 60 days, (e) RA at 60days, and (f) PS at 60 days

**
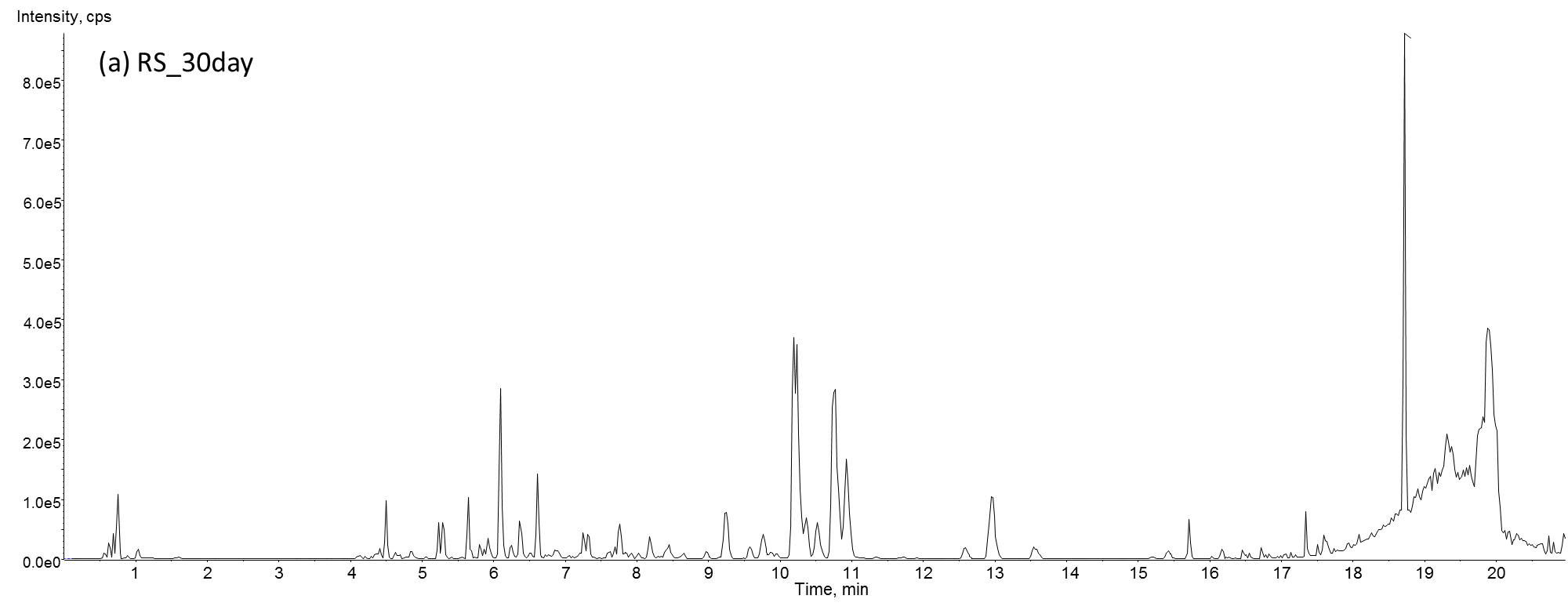
**

**
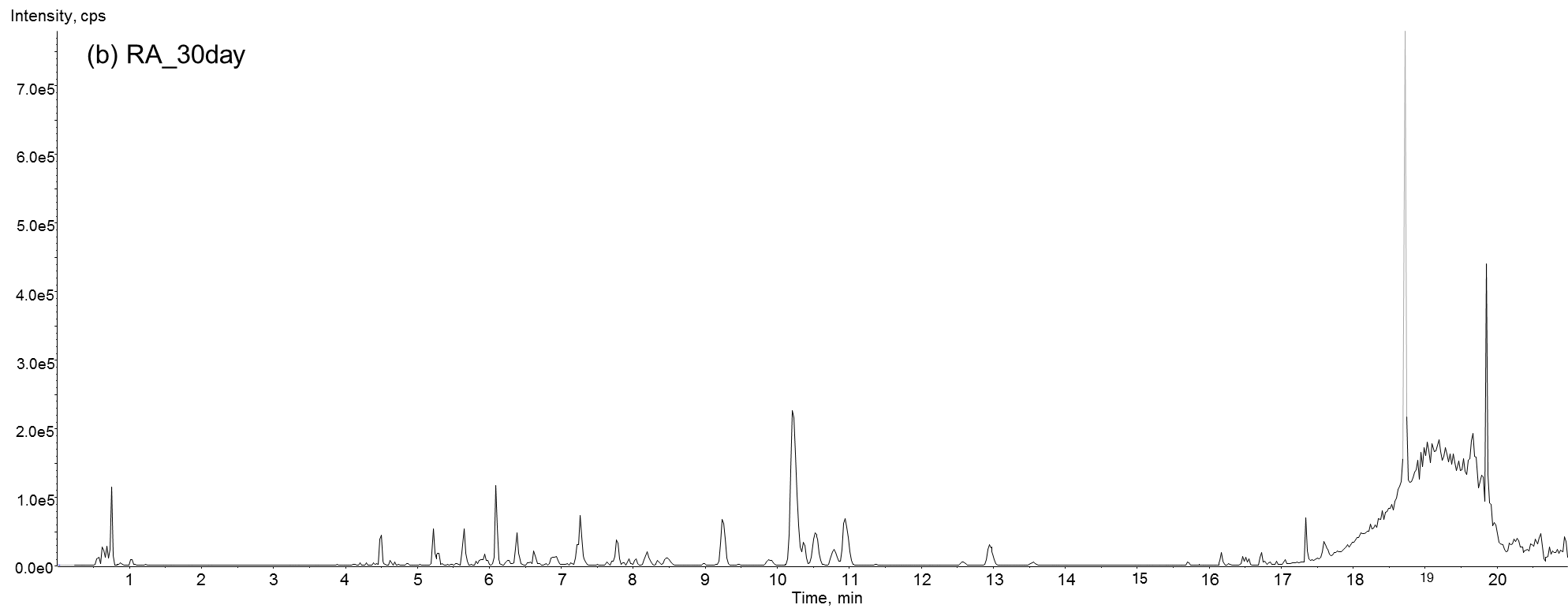
**

**
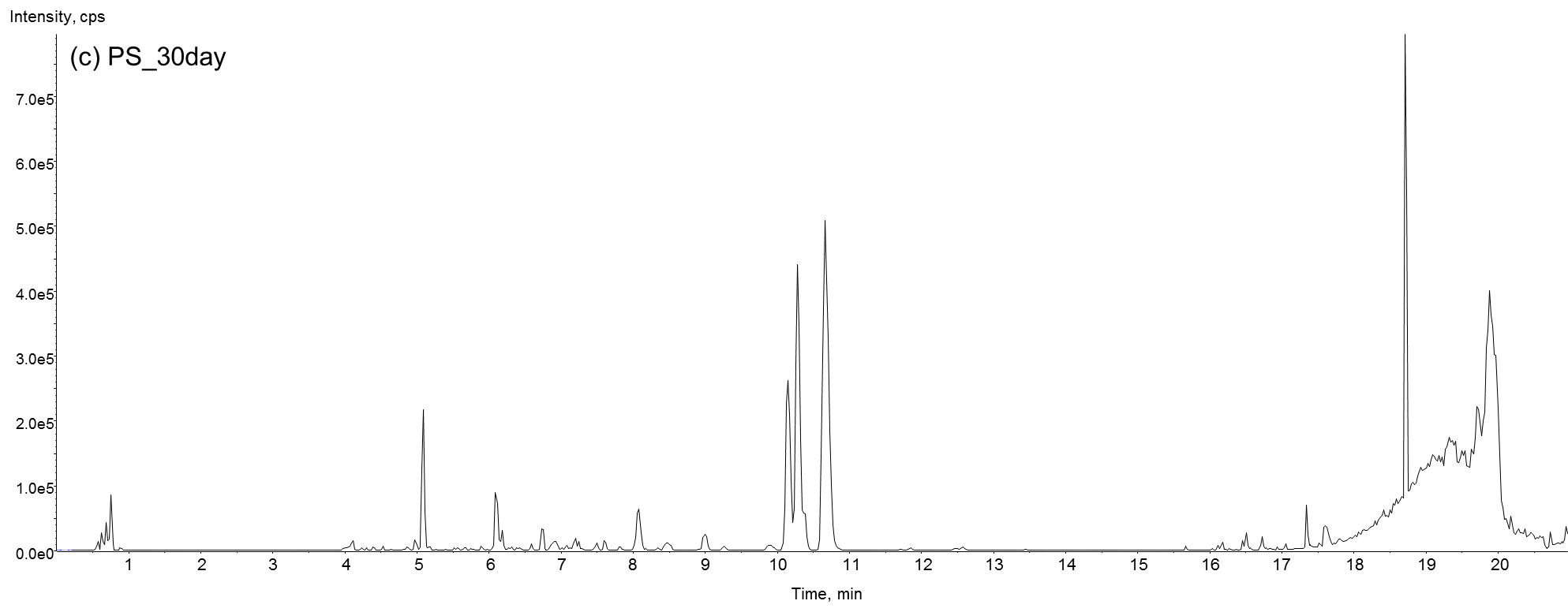
**

**
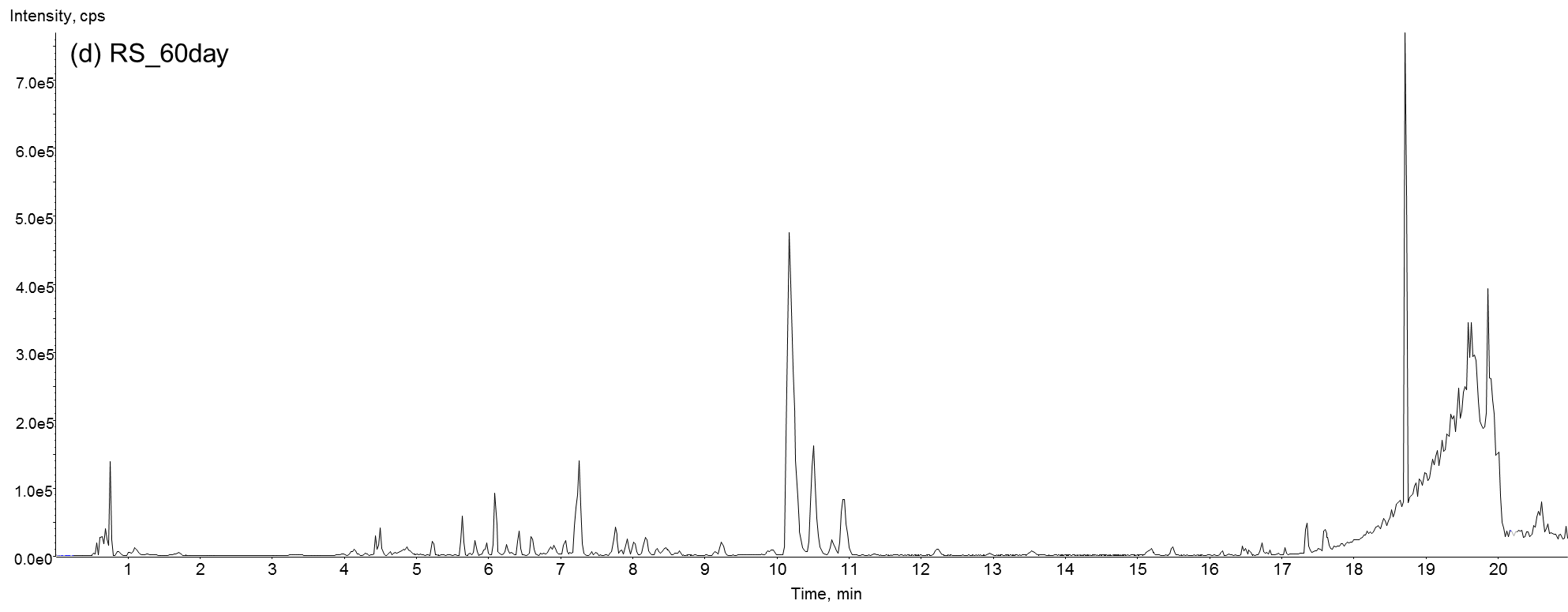
**

**
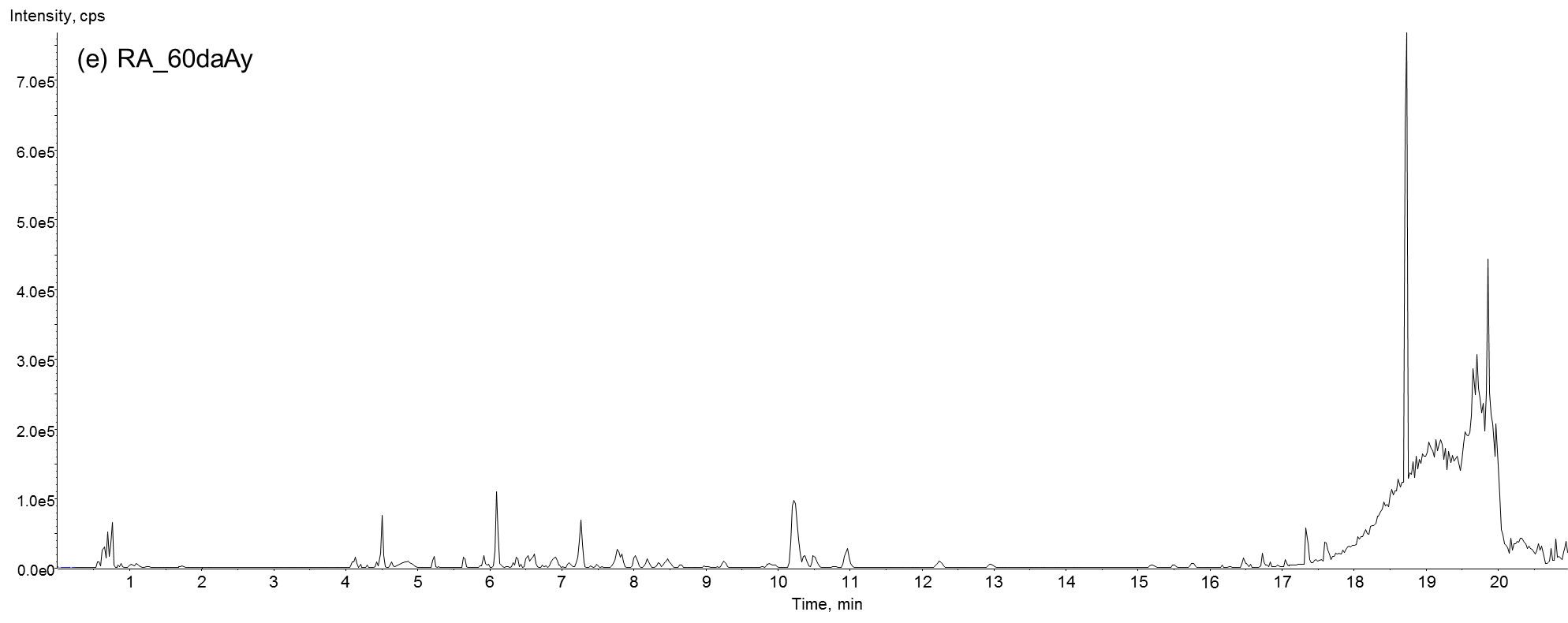

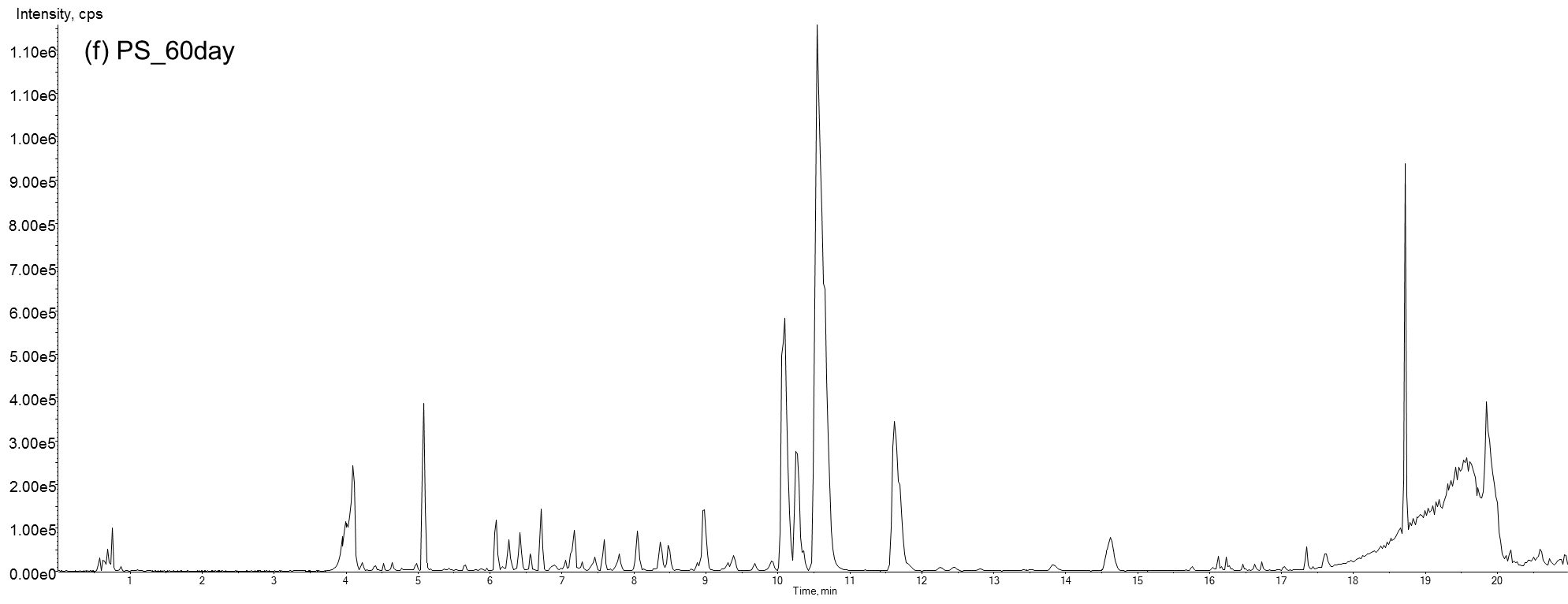
**

**Table S2**. Identification of alkaloids authentically or tentatively in aerial parts of the cultivated *P. rhoeas* and *P. somniferum* at different growing stages

| **Peak no.** | **Name** | | **Detected sample** | | | | | | | | | **Identification  (ID) by** |
| --- | --- | --- | --- | --- | --- | --- | --- | --- | --- | --- | --- | --- |
|  |  |  | **RS** | | | **RA** | | | **PS** | | |  |
|  |  |  | **30** | **60** | **90** | **30** | **60** | **90** | **30** | **60** | **90** |  |
|  |  |  | **cultivation periods (days)** | | | | | | | | |  |
| 1 |  | DL-Demethylcoclaurine | O | O | O | O | O | O | O | O | O | *1 |
| 2 |  | Coclaurine | O | O | O | O | O | O | O | O | O | *1 |
| 3 |  | Tetrahydropapaverine | — | — | — | — | — | — | O | O | O | *1 |
| 4 & 6 |  | Reticuline | O | O | O | O | O | O | O | O | O | *1 |
| 5 |  | Corytuberine | O | O | O | O | O | O | O | O | O | *1 |
| 7 |  | Tetrahydrocolumbamine | O | O | — | — | — | — | O | O | O | *1 |
| 8 |  | Scoulerine | — | — | — | — | — | — | O | O | O | *1 |
| 9 |  | L-Tetrahydropalmatine | — | — | O | — | — | — | O | O | O | *1 |
| 10 |  | Tetrahydroberberine (canadine) | — | — | O | — | — | — | O | O | O | *1 |
| 11 |  | Berberine | — | — | O | — | — | — | — | O | O | *1 |
| 12 |  | Stylopine | O | O | O | O | O | O | O | O | O | *1 |
| 13 |  | Dihydrosanguinarine | O | O | O | O | O | O | O | O | O | *1 |
| 14 |  | Sanguinarine | — | O | — | — | — | — | — | — | — | *1 |
| 15 |  | Protopine | O | O | O | O | O | O | O | O | O | *1 |
| 16 |  | Allocryptopine | — | — | — | — | — | — | O | O | O | *1 |
| 17 |  | Chelidonine | — | — | — | — | — | — | — | — | — | *1 |
| 18 |  | Dihydroberberine | — | — | — | — | — | — | — | — | — | *1 |
| 19 |  | Tyramine | O | O | O | O | O | O | O | O | O | *2 & 3 |
| 20 |  | Dopamine | O | O | O | O | O | O | O | O | O | *2 & 3 |
| 21 |  | 4-Hydroxyphenylacetaldehyde | O | O | O | O | O | O | O | O | O | * 3 |
| 22 |  | Morphine | — | — | — | — | — | — | O | O | O | * 3 |
| 23 |  | Mecambrine | — | — | — | — | — | — | O | O | O | * 3 |
| 24 |  | Codeine | — | — | — | — | — | — | O | O | O | * 3 |
| 25 |  | (S)-N-Methylcoclaurine | O | O | O | O | O | O | O | O | O | * 3 |
| 26 |  | Armepavine | O | O | O | O | O | O | O | O | O | * 3 |
| 27 |  | (S)-3'-Hydroxy-N-methylcoclaurine | O | O | O | O | O | O | O | O | O | * 3 |
| 28 |  | (S)-Cheilanthifoline | O | O | O | O | O | O | — | O | O | * 3 |
| 29 |  | Papaverine | — | — | — | — | — | — | O | O | O | * 3 |
| 30 |  | Cryptopine | O | O | O | O | O | O | O | O | O | * 4 |
| 31 |  | Noscapine | — | — | O | — | O | — | O | O | O | * 4 |
| 32 |  | 4-Hydroxyphenylpyruvate | O | O | O | O | O | O | O | O | O | † |
| 33 |  | Codeinone | O | — | — | — | — | — | — | — | — | † |
| 34 |  | Morphine N-oxide | — | — | — | — | — | — | O | O | O | † |
| 35 |  | Flavinantine | — | — | — | — | — | — | — | O | O | † |
| 36 |  | 8,14-dihydroflavinantine  (or Salutaridinol) | O | O | O | O | O | O | O | O | O | † |
| 37 |  | (S)-cis-N-Methylstylopine | O | O | O | O | O | O | — | — | — | † |
| 38 |  | Isocorydine | O | O | O | O | O | O | O | — | — | † |
| 39 |  | Pseudoprotopine | O | O | O | — | O | O | — | — | — | † |
| 40 |  | Amurensinine N-oxide A  (or amurensinine N-oxide B) | O | O | O | O | O | O | O | O | O | † |
| 41 |  | Rheagenine  (or isorheagenine) | O | O | O | O | O | O | — | — | — | † |
| 42 |  | Rhoeadine  (or isorhoeadine) | O | O | O | O | O | O | — | — | — | † |
| 43 |  | Glaucamine  (or isoglaucamine) | O | O | O | O | O | O | — | — | — | † |
| 44 |  | Coptisine | O | O | — | O | O | — | O | O | — | † |
| 45 |  | Unknown M1 | O | O | O | O | O | O | O | O | O | ‡ |
| 46 |  | Unknown M2 | — | — | — | — | — | — | — | O | O | ‡ |
| 47 |  | Unknown M3 | O | O | O | O | — | O | — | — | O | ‡ |
| 48 |  | Unknown M4 | O | O | O | O | O | O | — | — | — | ‡ |
| 49 |  | Unknown M5 | O | O | O | O | O | O | O | O | O | ‡ |
| 50 |  | Unknown M6 | O | O | O | O | O | O | O | O | O | ‡ |
| 51 |  | Unknown M7 | — | O | O | — | O | O | — | — | — | ‡ |
| 52 |  | Unknown M8 | O | O | O | O | O | O | O | O | O | ‡ |
| 53 |  | Unknown M9 | O | — | O | — | — | — | — | — | — | ‡ |
| 54 |  | Unknown M10 | O | O | O | O | O | O | — | — | — | ‡ |
| 55 |  | Unknown M11 | O | O | O | O | O | O | — | — | — | ‡ |

*1: authentic standard
*2: in-house MS/MS library (SCIEX)
*3: Metlin
*4: MS bank

† : isotope patters and literatures
‡ : unknown metabolites, ms/ms not mateched with known alkaloids in Metlin

O: Presence; —: Absence.


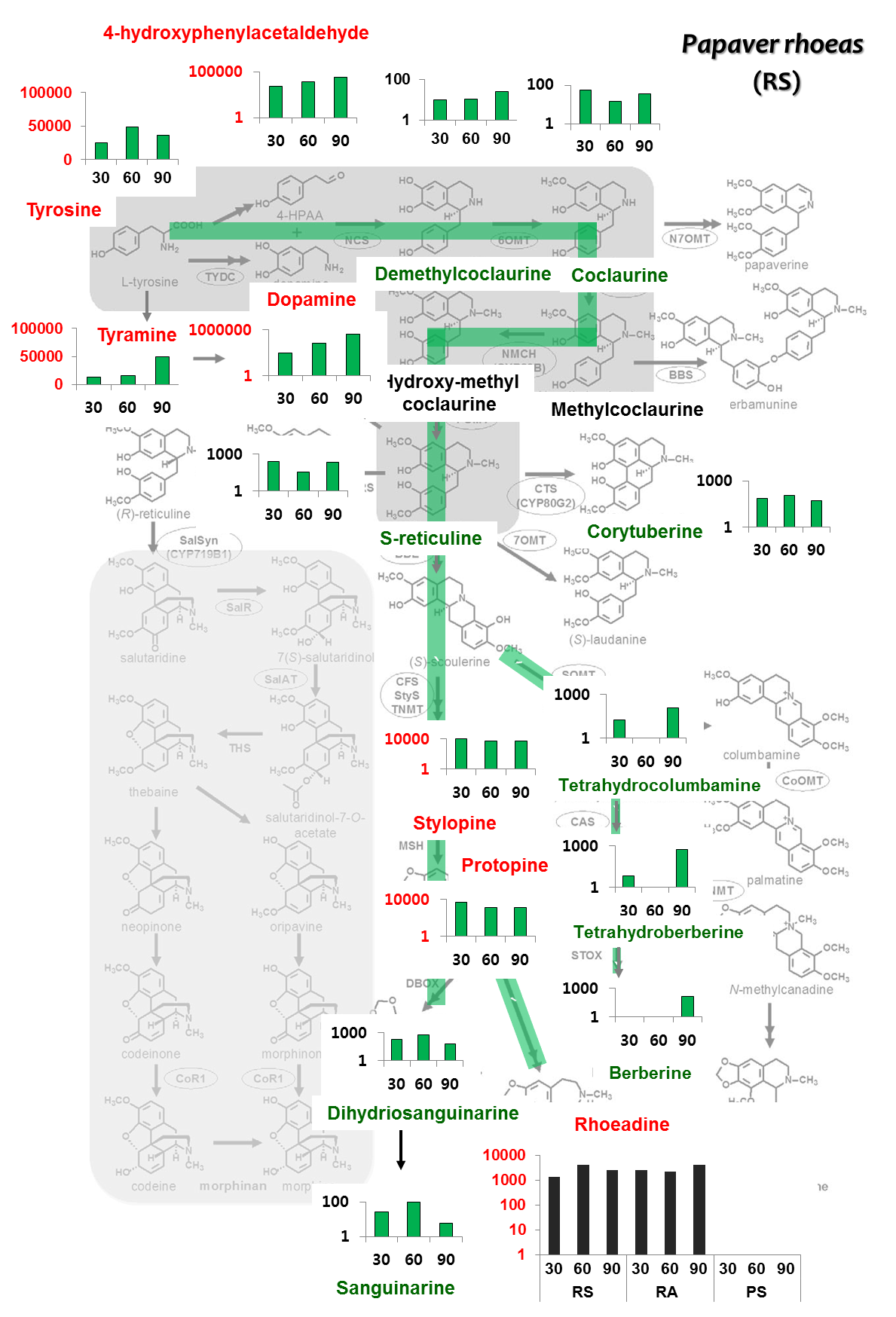


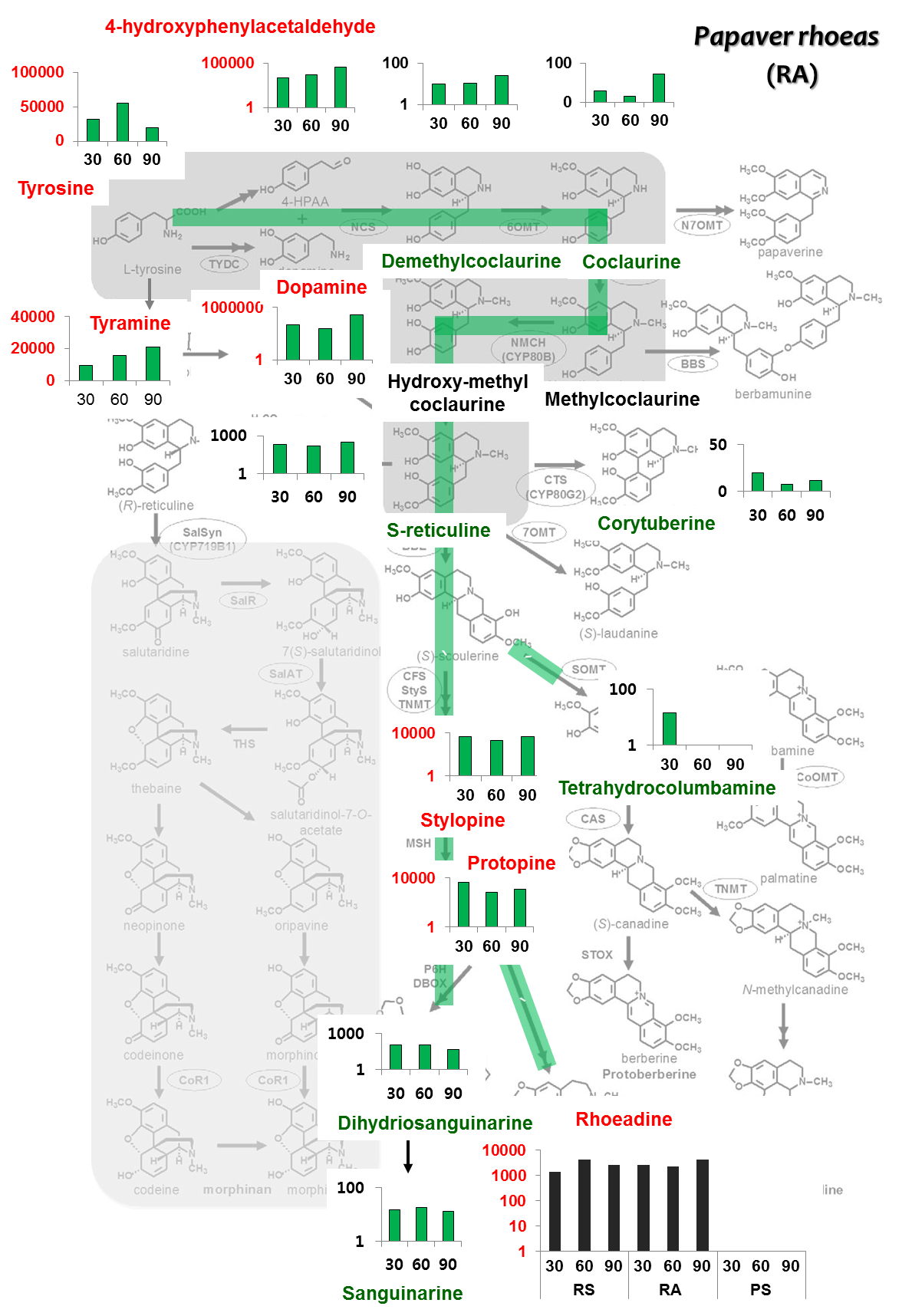


**Figure S4.** Metabolites (colored or bold text) from *Papaver rhoeas* (RS and RA) identified in benylisoquinoline alkaloid (BIA) biosynthesis pathways [*Phytochemistry 70 (2009)*]. The ratio is the peak intensity of a metabolite in a sample that was divided by the peak intensity of a blank sample and expressed in a logarithmic scale.

RS: Shiri *P. rhoeas* seeds from Moscow, Russia

RA: WS2981 *P. rhoeas* seeds from Hoengseong in the Province of Gangwon, South Korea *1696–1707*.
